# The Contribution of Alu Elements to Mutagenic DNA Double-Strand Break Repair

**DOI:** 10.1371/journal.pgen.1005016

**Published:** 2015-03-11

**Authors:** Maria E. Morales, Travis B. White, Vincent A. Streva, Cecily B. DeFreece, Dale J. Hedges, Prescott L. Deininger

**Affiliations:** 1 Tulane Cancer Center and Department of Epidemiology, Tulane University Health Sciences Center, New Orleans, Louisiana, United States of America; 2 Department of Biology, Xavier University of Louisiana, New Orleans, Louisiana, United States of America; 3 Division of Human Genetics, Department of Internal Medicine, The Ohio State University, Columbus, Ohio, United States of America; Duke University, United States of America

## Abstract

Alu elements make up the largest family of human mobile elements, numbering 1.1 million copies and comprising 11% of the human genome. As a consequence of evolution and genetic drift, Alu elements of various sequence divergence exist throughout the human genome. Alu/Alu recombination has been shown to cause approximately 0.5% of new human genetic diseases and contribute to extensive genomic structural variation. To begin understanding the molecular mechanisms leading to these rearrangements in mammalian cells, we constructed Alu/Alu recombination reporter cell lines containing Alu elements ranging in sequence divergence from 0%-30% that allow detection of both Alu/Alu recombination and large non-homologous end joining (NHEJ) deletions that range from 1.0 to 1.9 kb in size. Introduction of as little as 0.7% sequence divergence between Alu elements resulted in a significant reduction in recombination, which indicates even small degrees of sequence divergence reduce the efficiency of homology-directed DNA double-strand break (DSB) repair. Further reduction in recombination was observed in a sequence divergence-dependent manner for diverged Alu/Alu recombination constructs with up to 10% sequence divergence. With greater levels of sequence divergence (15%-30%), we observed a significant increase in DSB repair due to a shift from Alu/Alu recombination to variable-length NHEJ which removes sequence between the two Alu elements. This increase in NHEJ deletions depends on the presence of Alu sequence homeology (similar but not identical sequences). Analysis of recombination products revealed that Alu/Alu recombination junctions occur more frequently in the first 100 bp of the Alu element within our reporter assay, just as they do in genomic Alu/Alu recombination events. This is the first extensive study characterizing the influence of Alu element sequence divergence on DNA repair, which will inform predictions regarding the effect of Alu element sequence divergence on both the rate and nature of DNA repair events.

## Author Summary

DNA double-strand breaks (DSBs) are a highly mutagenic form of DNA damage that can be repaired through one of several pathways with varied degrees of sequence preservation. Faithful repair of DSBs often occurs through gene conversion in which a sister chromatid is used as a repair template. Unfaithful repair of DSBs can occur through non-allelic homologous or homeologous recombination, which leads to chromosomal abnormalities such as deletions, duplications, and translocations and has been shown to cause several human genetic diseases. Substrates for these homologous and homeologous events include Alu elements, which are approximately 300 bp elements that comprise ~11% of the human genome. We use a new reporter assay to show that repair of DSBs results in Alu-mediated deletions that resolve through several distinct repair pathways. Either single-strand annealing (SSA) repair or microhomology-mediated end joining occurs ‘in register’ between two Alu elements when Alu sequence divergence is low. However, with more diverged Alu elements, like those typically found in the human genome, repair of DSBs appears to use the Alu/Alu homeology to direct non-homologous end joining in the general vicinity of the Alu elements. Mutagenic NHEJ repair involving divergent Alu elements may represent a common repair event in primate genomes.

## Introduction

DNA double-strand breaks (DSBs) are the most dangerous type of DNA damage due to their tendency to lead to chromosomal rearrangements, a hallmark of tumorigenesis, when they are repaired [1]. One way in which chromosomal rearrangements occur in DSB repair is the use of non-allelic recombination between repetitive elements (reviewed in [2]), which comprise a large portion of the human genome [3]. Alu elements have amplified over the past 65 million years and occupy about 11% of the human genome, with well over one million copies [3]. The majority of identified Alu elements diverge 4%-20% from the consensus [3]. Despite this level of sequence divergence, Alu elements represent a major source of sequence homology in the human genome and contribute to genomic instability that arises from mutagenic recombination between these elements [4,5]. Furthermore, Alu/Alu recombination is estimated to cause as many as 0.5% of all new genetic diseases and is responsible for mutations that contribute to human cancers [4–6].

Recombination between Alu elements can occur through completely identical (homologous) Alu sequences, but most events involve Alu elements with approximately 20% mismatch relative to one another (homeologous), which reflects the average sequence divergence of proximal elements. For the purposes of this study, we will refer to any DNA repair event that occurs through either homologous or homeologous recombination between two non-allelic Alu elements and generates a single chimeric, Alu element, as Alu/Alu recombination. We consider only genomic deletions in this study because our reporter system only detects DNA repair products that result in a deletion. A number of different pathways can give rise to these Alu/Alu deletions, including single-strand annealing (SSA) repair that may predominate when there are high levels of homology, and mechanisms such as microhomology-mediated end joining (MMEJ) where the microhomology happens to be ‘in register’ between the two Alu elements, allowing formation of a single chimeric Alu element [7]. This latter mechanism is more likely when there is significant sequence divergence between the Alu elements and has also been referred to as “micro-SSA” [7].

Repair of a DNA DSB via the SSA pathway occurs between two direct-repeat elements on the same DNA duplex, and results in deletion of the intervening genetic material. It is initiated by nuclease-mediated resection of the DNA ends at the break to generate 3’-single-stranded DNA (ssDNA) overhangs that expose the repeat elements for base pairing (reviewed in [8]). Rad52 binds ssDNA and mediates both a search for sequence homology and DNA strand annealing in *cis* to form a DNA duplex [9]. The annealed DNA intermediate can be processed by endonucleolytic cleavage of the 3’-ssDNA overhangs, DNA polymerase-mediated extension of the new 3’ ends, and ligation of the completely extended product, which results in the deletion of the sequences between the repeat elements [10]. If mismatched DNA bases are present in the annealed duplex, helicases may be recruited to unwind the mismatched duplex in a process termed heteroduplex rejection [11,12]. Alternatively, molecules that escape heteroduplex rejection may be replicated prior to repair or subjected to mismatch repair (MMR) prior to replication [11].

MMEJ is a form of alternative non-homologous end joining (also called alt-NHEJ) [13]. Like SSA, MMEJ requires DNA resection which allows exposed DNA sequences to base pair at microhomologies (5–25 nucleotides) and results in a deletion of the intervening sequence [13]. This error-prone method of repair occurs independent of the canonical NHEJ factors Ku and DNA-PKcs, and genetic analyses in yeast reveal that MMEJ is also Rad52-independent, which distinguishes it from SSA repair [14]. Because the MMEJ repair outcome always results in genomic deletions, MMEJ is thought to significantly contribute to genomic instability [13]. Homeologous Alu elements present ample opportunity for MMEJ repair via short microhomologies that are ‘in register’ between the two elements to create a chimeric Alu element that looks essentially identical to the product of a SSA recombination [7].

In this study, we characterized the impact of sequence divergence on the repair of DNA DSBs between Alu elements by Alu/Alu recombination and a subset of NHEJ events using an Alu/Alu recombination reporter cassette. For the first time, we have extensively quantified the impact of homeologous (similar but not identical) sequences on the rate of intrachromosomal Alu/Alu recombination. Using our Alu/Alu recombination reporter system, we observed a striking decrease in the rate of intrachromosomal Alu/Alu recombination when the sequence divergence between the Alu elements used for the repair increased. Additionally, as sequence divergence increased, there was a significant increase in NHEJ repair in the vicinity of the Alu elements that did not result in Alu/Alu recombination. These heterogeneous NHEJ repair events appear to be stimulated by the overall Alu homeology, and therefore represent a new alternative influence on the NHEJ pathway that we have termed homeology-influenced NHEJ (HI-NHEJ). These results will aid in predicting which regions of the human genome may be particularly susceptible to Alu-mediated deletions and will give greater insight into genomic recombination hot spots that lead to human disease.

## Results

### A reporter system to detect and characterize Alu/Alu recombination

**Fig 1:**
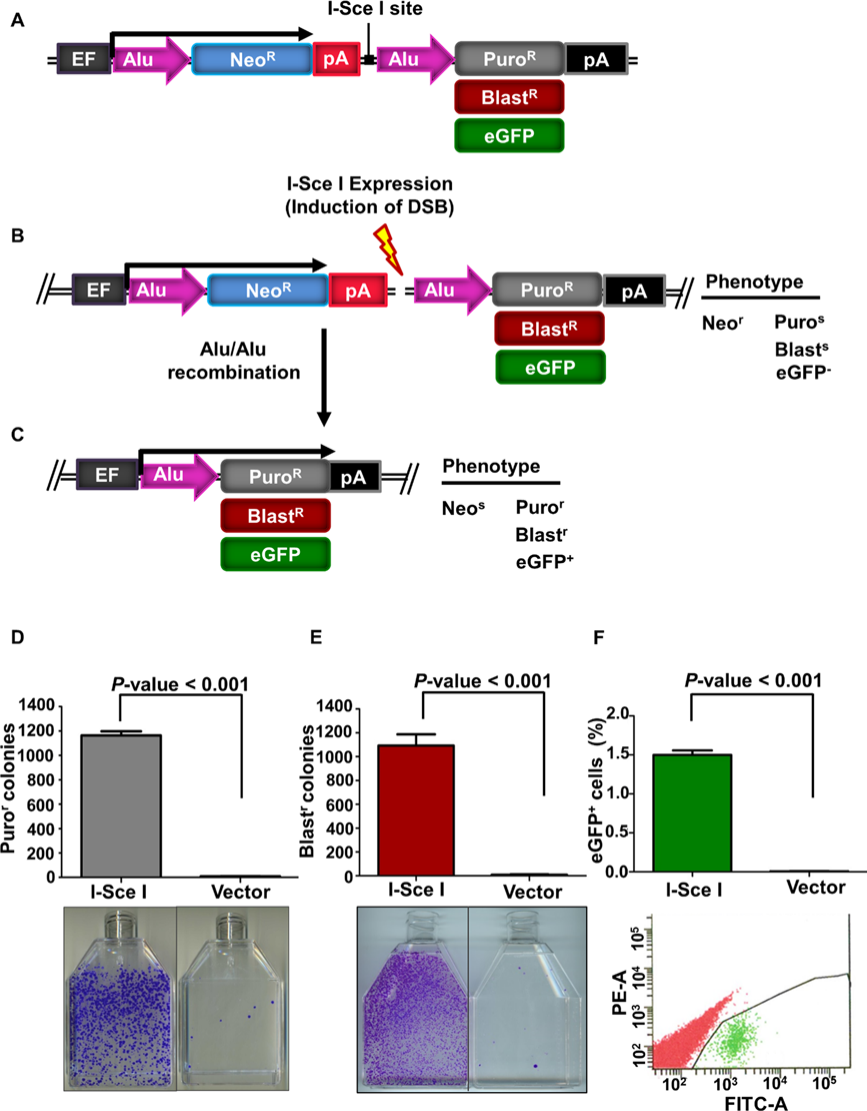
The Alu/Alu recombination system. **(A)** Schematic of the Alu/Alu recombination reporter. Cells harboring the reporter were neomycin resistant (neo^r^) and sensitive to puromycin (puro^s^) or blasticidin (blast^s^) or eGFP negative (eGFP^-^), depending on the reporter cassette used (AARP, AARB or AARG). Cells were grown in the presence of neomycin to eliminate cells that underwent spurious recombination before induction of DNA double-strand breaks (DSBs). DSBs were induced by transfection of an I-SceI endonuclease expression vector. **(B)** The reporter cassette was inserted using the Flp-In system (see S1 Fig, Materials and Methods) into a single genomic location in each cell line, which allows variants of the vector (i.e. different Alu elements) to be inserted in the same chromosomal location. **(C)** Repair of an I-SceI-induced DNA DSB may result in a deletion of the sequence between the two Alu elements after which the cell becomes neomycin sensitive (neo^s^) and express the puromycin resistance (puro^R^), blasticidin resistance (blast^R^) or eGFP (eGFP^+^) gene. (**D and E**) I-SceI-induced DNA DSBs in the AARP **(D)** and AARB **(E)** HEK293FRT cells result in Alu/Alu recombination. Cells were transiently transfected with either I-SceI expression vector or empty vector (pUC19) control. Puromycin resistant (puro^r^) or blasticidin resistant (blast^r^) colonies were counted and are shown graphically. Error bars denote standard error and statistical significance was shown using one-way ANOVA. Below the graphs are representative flasks showing puro^r^ or blast^r^ colonies following I-SceI-induced DNA DSBs. (F) I-SceI-induced DNA DSBs in 0%-AARG HEK293FRT cells result in eGFP^+^ cells. The percentage of eGFP^+^ cells are shown graphically. Error bars represent standard error and statistical significance was shown using Student’s t-test. Representative flow plots from fluorescence activated cell sorting (FACS) analysis following transfections with I-SceI or empty vector pUC19 are shown below the graphs.

**Fig 2:**
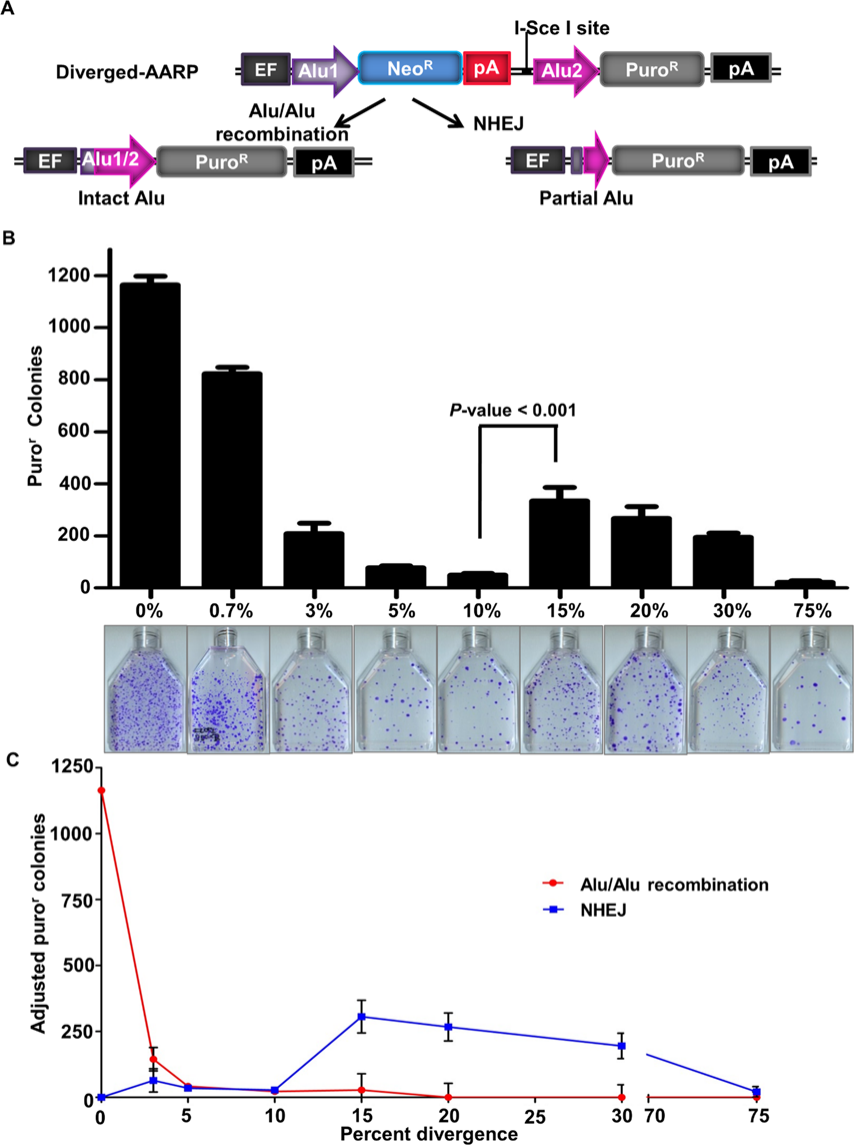
The effect of sequence divergence on Alu/Alu recombination. **(A)** Schematic of AARP cassettes with varying degrees of sequence divergence. Stably integrated diverged-AARP HEK 293FRT cell lines were created using the Flp-In system (see S1 Fig, Materials and Methods) and were transfected with an I-SceI expression vector to induce DNA DSBs. In addition to Alu-mediated deletions resulting from Alu/Alu recombination, the AARP reporter detects a subset of non-homologous end joining (NHEJ) products that result in large deletions. **(B)** The average number of puro^r^ colonies is plotted for the indicated AARP HEK293FRT cells tested (Table 1). Data from at least three independent experiments using at least three independently isolated clones for each AARP cell line are averaged. The average of independent experiments using two scrambled-sequence (Scr1- and Scr2-AARP) HEK293FRT cell lines is shown for the 75% data point (S5 Fig and Table 1). Error bars denote standard error and statistical significance is shown using one-way ANOVA. Representative T75 culture flasks of stained puro^r^ colonies are shown below the graphs. **(C)** Puro^r^ colony numbers are adjusted and plotted according to the percentage of Alu/Alu recombination or NHEJ for the indicated AARP HEK293FRT cells tested as determined by PCR and sequence analysis of DNA repair products from isolated puro^r^ colonies.

We have designed a novel Alu/Alu recombination reporter cassette to quantitatively assess deletions caused by Alu elements. The basic construct consists of an human elongation factor 1α (EF1α) promoter upstream of two Alu sequences separated by approximately 1100 bp of sequence encoding a neomycin resistance (neo^R^) gene, a polyadenylation signal (pA), and an I-SceI endonuclease cleavage site to generate a DNA DSB between the two Alu elements (Fig. 1A). Maintaining the cells under neomycin selection eliminated almost all background Alu/Alu recombination that occurred prior to experimental treatment. A puromycin resistance (puro^R^) gene downstream of the Alu sequences is not initially expressed because it is separated from the EF1α promoter by the two Alu sequences, the neo^R^ gene, and a polyadenylation signal (Fig. 1A and B). We designated this construct Alu/Alu recombination puromycin (AARP). If DNA repair results in a deletion of the sequence between the two Alu elements, the cells lose neo^R^, and the puro^R^ gene comes into proximity of the EF1α promoter to confer puro^R^ (Fig. 1C). Each component of the AARP reporter cassette is flanked with unique restriction enzyme sites to allow for rapid interchange of components in order to explore potential modulators of Alu/Alu recombination as well as different reporter genes (Fig. 1C and S1 Fig). In addition to recovering Alu/Alu recombination events, the AARP reporter system also can detect a subset of non-homologous end joining (NHEJ) events that result in ~1.0–1.9 kb deletions of the sequence near the I-SceI cleavage site but do not recreate a single Alu element (Fig. 2A). For simplicity, we will refer to these variable-length deletions as NHEJ events.

**Table 1:** The average puromycin resistant (puro^r^) colonies and repair pathway contribution for AARP HEK293FRT cells.

Alu sequence divergence in AARP^a^	Average Number of puro^r^ Colonies^b^	Puro^r^ colonies fold decrease relative to 0%	Alu/Alu recombination (%)^c^	NHEJ (%)^d^
0%	1164 (±34)	—	100	0
0.7%	822 (±25)	1.4	NA^e^	NA^e^
3%	209 (±39)	6	69	31
5%	77 (±08)	15	55	45
10%	50 (±06)	23	44	56
15%	334 (±52)	3	9	91
20%	267 (±46)	4	0	100
30%	195 (±16)	6	0	100
75% (scrambled sequence)^f^	21 (±6)	55	0	100

a) S4 Fig includes the sequences of the specific Alu elements in each construct.

b) Colony numbers per 10^6^ cells are reported with standard error.

c) Alu/Alu recombination percentage was calculated as the number of repair junctions containing an intact Alu as a proportion of total number of sequenced repair junctions.

d) NHEJ percentage was calculated as the number of repair junctions containing a non-intact Alu as a proportion of total number of sequenced repair junctions.

e) Data could not be calculated because PCR and sequence analysis of repair products was not performed for 0.7%-AARP HEK293FRT cells.

f) The average of independent experiments using two scrambled-sequence AARP HEK293FRT cell lines (Scr1-AARP and Scr2-AARP; S5 Fig).

### Generation of stable Alu/Alu recombination reporter cell lines

To generate stable cell lines carrying only a single copy of our Alu/Alu recombination reporter cassette and to avoid variability from random genomic integration of our vector, we used the Flp-In system (Life Technologies, Grand Island, NY) to direct integration into a FRT site at a single, chromosome-specific location (S1 Fig, Materials and Methods). We stably integrated AARP into the genome of HEK293FRT cells (HEK293-based Flp-In cells; Life Technologies), and isolated individual hygromycin and neomycin resistant clones. This approach allowed us to integrate vector variants into the same site to directly compare the effects of various parallel variations of our reporter cassette on Alu/Alu recombination, in an isogenic manner. To confirm AARP was integrated as a single copy, we used droplet digital polymerase chain reaction (ddPCR), a powerful tool for precisely evaluating the copy number of genomic targets [15–17]. Using ddPCR and the RPP30 housekeeping gene as a copy-number standard, we confirmed that our AARP reporter cassette inserted as a single genomic copy into our parental HEK293FRT cell line using (S2 Fig).

Three independent FRT-integrated cell clones for each Alu variant were selected and transiently transfected with an I-SceI endonuclease expression vector to induce formation of DNA DSBs between the two Alu elements. Fourteen days later, puromycin resistant (puro^r^) colonies were counted to assess the level of recombination. Each of the three independent cell clones testing Alu/Alu recombination between homologous Alu elements (0%-AARP HEK293FRT cells) gave comparable numbers of puro^r^ colonies after I-SceI expression, with an average of 1000 colonies per T75 culture flask (Fig. 1D). In the absence of I-SceI expression, puro^r^ colonies were detected at very low numbers, which indicates that almost all of the experimental puro^r^ colonies arose from repair of I-SceI-generated DNA DSBs.

In order to make this Alu/Alu recombination reporter available for use in puro^r^ cells, we replaced the puro^R^ gene with a blasticidin resistance (blast^R^) gene and green fluorescent protein (eGFP^+^) to create Alu/Alu recombination blasticidin (AARB) and Alu/Alu recombination eGFP (AARG), respectively. These reporters produced similar results to AARP in HEK293FRT cells (Fig. 1E and 1F). We also performed Alu/Alu recombination experiments in the following Flp/In cell lines: baby hamster kidney (BHKFRT), mouse embryonic fibroblast (NIH-3T3FRT), and Chinese hamster ovary (CHOFRT) (Life Technologies). As expected, cells transfected with an I-SceI endonuclease expression vector resulted in significant numbers of puro^r^ or blasticidin resistant (blast^r^) colonies, which indicates recombination (S3 Fig). To confirm that the puro^r^ or blast^r^ colonies arose from the expected recombination within our Alu/Alu recombination reporter cassette, we performed PCR and sequence analysis of I-SceI-induced DNA repair products from isolated puro^r^ AARP or blast^r^ AARB colonies was performed (Materials and Methods, S2 Table). Sequence analysis confirmed that puro^r^ AARP or blast^r^ AARB colonies generated the expected Alu/Alu recombination product (Fig. 1C) between the Alu elements in the Alu/Alu recombination reporter cassette.

### The effect of Alu element sequence divergence on DNA deletion frequency

Because Alu elements of various levels of divergence exist throughout the human genome [3], we sought a quantitative assessment of the effect of sequence divergence on the rate of Alu/Alu recombination. AARP constructs containing evenly spaced sequence divergence at 0.7%, 3%, 5%, 10%, 15%, 20%, and 30% between the two Alu elements were created and integrated as single-copy reporters in HEK293FRT cells as described in Materials and Methods (Fig. 2A and S4 Fig). To induce DNA repair events, AARP HEK293FRT cells were transfected with an I-SceI endonuclease expression vector. The introduction of as little as 0.7% sequence divergence between the AARP Alu elements resulted in a significant reduction in puro^r^ colonies relative to homologous Alu elements (Fig. 2B, S1 Table and S4 Table), which indicates that even small degrees of sequence divergence can reduce the efficiency of homology-directed DSB repair. Further reduction in puro^r^ colonies was observed in a sequence-divergence-dependent manner for diverged-AARP constructs up to 10%. We observed a significant increase in puro^r^ colonies when the sequence divergence between the Alu elements in AARP was increased to 15%, 20%, and 30%. However, experiments with two different scrambled-sequence AARP constructs (75% divergence) resulted in a significant reduction in overall colony number relative to 15%-30% sequence divergence (Fig. 2B, Table 1, and S5 Fig). This observation led us to hypothesize that the increase in puro^r^ colonies with 15%-30% sequence divergence between Alu elements relative to 5%-10% is due to a shift in the type of DNA repair responsible for resolution of the DNA DSB (see below).

To identify the nature of the deletions caused by our I-SceI-induced DSBs, we collected genomic DNA from multiple isolated puro^r^ colonies, and subjected it to PCR and sequence analysis (Fig. 2A and S6 Fig). When homologous Alu elements were tested (0%-AARP), repair occurred exclusively through Alu/Alu recombination. However, because of the sequence identity of the Alu elements, the exact recombination junction within the Alu element could not be discerned. With homeologous Alu elements, we were able to map the recombination junction of the Alu/Alu recombination products as well as identify NHEJ repair products that did not result in a single chimeric Alu element. We observed a major drop in the Alu/Alu recombination rate as the divergence between Alu elements in AARP cells increased from 0% to 30%. As hypothesized, the increase in puro^r^ colonies as sequence divergence between Alu elements increased from 10% to 15%, 20%, or 30% correlated with a shift in DNA repair from Alu/Alu recombination to variable-length NHEJ (indicated by recovery of partial Alu sequences rather than intact Alu sequences). Repair via NHEJ was responsible for the vast majority of products that involve two Alu elements with ≥15% sequence divergence (Fig. 2C, S7 Fig, and S8 Fig). The dramatic decrease in puro^r^ colonies as sequence divergence between the Alu elements increased from 30% to 75% (scrambled sequences) indicated that there is a sequence homology-driven component to the NHEJ DSB repair that makes use of homeologous Alu sequences to stimulate repair in the vicinity of the Alu elements (Fig. 2C, S7 Fig, S8 Fig, and Table 1).

### The distribution of Alu/Alu recombination junctions

**Fig 3:**
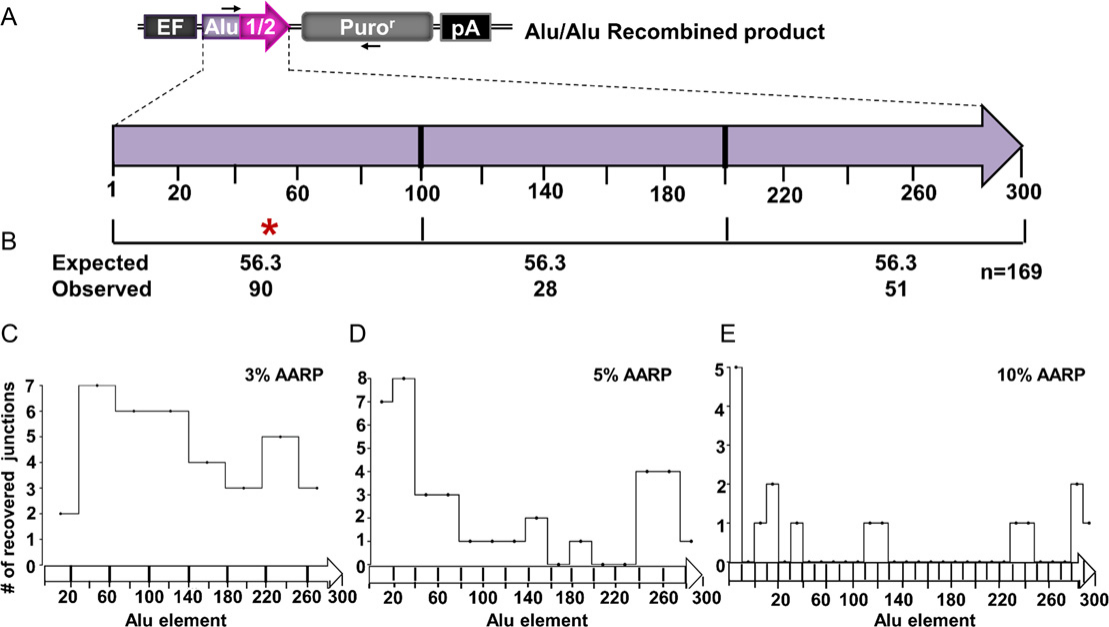
The distribution of Alu/Alu recombination junctions in AARP HEK293FRT cells. **(A)** A schematic of the Alu/Alu recombination product indicates, in purple, a 5’ section of Alu1 (containing sequence divergence relative to Alu2) and, in pink, Alu 2 (Alu Ya5 consensus sequence). Arrows represent PCR primers used to amplify Alu/Alu recombination products (S2 Table). **(B)** The distribution of Alu/Alu recombination junctions in 3%-, 5%-, and 10%-AARP HEK293FRT cells as determined by PCR and sequence analysis of DNA repair products from isolated puro^r^ colonies. The Alu/Alu recombination product is divided into three segments of equal length (100 bp), which each contain the same extent of sequence divergence. The number of Alu/Alu recombination junctions expected and observed in each 100 bp interval is shown. An asterisk (*) marks an interval in which p<0.05 significance as determined by a chi-square test for observed vs. expected. **(C-E)** Alu/Alu recombination junctions mapped individually for the 3%- **(C)**, 5%- **(D)**, and 10%- **(E)** AARP HEK293FRT cells. The Alu/Alu recombination product was divided into segments of equal length according to the intervals of homology in which the Alu/Alu recombination junction can be mapped for each diverged AARP construct. The number of Alu/Alu recombination junctions observed is plotted on the y-axis for each interval of homology.

Several studies have shown that Alu/Alu recombination junctions naturally show a preference within the 5’ half of the Alu element [18–20]. To determine if Alu/Alu recombination junctions in our system similarly cluster to a particular region of the participating Alu elements, we mapped the recombination junctions of169 Alu/Alu recombination events from diverged AARP HEK293FRT cells (Fig. 3A). The Alu/Alu recombination product (chimeric Alu element) was divided into three 100 bp segments and the number of observed Alu/Alu recombination events in each interval was compared to the expected count if the recombination events occurred at random throughout the participating Alu elements (Fig. 3B). We observed a statistically significant increase in the number of recombination junctions occurring within the first 100 bp of the Alu element when compared to the remaining portion of the Alu. This is consistent with previous reports of genomic Alu elements involved in Alu/Alu recombination that suggest the presence of preferential recombination in the 5’ end of Alu [18,21]. We next wanted to assess the distribution of the Alu/Alu recombination junctions in the 3%-AARP, 5%-AARP, and 10%-AARP HEK293FRT cells separately. While the junctions in 3%-AARP cells appear evenly distributed (Fig. 3C), there is a clear bias toward the first 100 bp of the Alu element in 5%-AARP and 10%-AARP cells (Fig. 3D and 3E). To determine if the preference for the formation of a recombination junction within the Alu element was mediated by the length of the region of perfect homology, we modified the 5%-AARP constructs to include two longer stretches of homology, 40 bp, either at each end (S9A Fig) or in the middle of the Alu element (S9B Fig) and a construct with 100 bp of homology in the middle of the Alu element (S9C Fig). Surprisingly, introduction of increased lengths of perfect sequence homology did not shift the Alu/Alu recombination junction preference from the first 100 bp of the Alu element (S9 Fig). Thus, although the overall sequence homology between Alu elements clearly contributes to the rate of Alu/Alu recombination, the actual resolution of the repair junction does not appear to be directed to regions of longer homology.

### The distribution of Alu/Alu NHEJ junctions

To determine breakpoint locations of NHEJ-mediated deletions, we analyzed DNA repair junctions isolated from AARP HEK293FRT puro^r^ colonies by PCR and sequencing. We found 36% of the NHEJ repair junctions were deletions between different locations in both Alu elements in the AARP reporter cassette (S7 Fig and S8 Fig). Of the NHEJ deletion events involving two Alu elements, 84% used microhomology between the two Alu elements during repair. Of the NHEJ repair events that did not involve both Alu elements at the repair junction, the majority used at least one Alu element and used microhomology elsewhere in the AARP reporter cassette 75% of the time. To further classify the NHEJ deletion products, we analyzed the size of the deletion caused by each NHEJ repair event in AARP HEK293FRT cells. We found Alu-mediated NHEJ deletions ranged from 897 bp to 1881 bp (S7 Fig and S8 Fig). However, due to the nature of our AARP reporter system, we were not able to detect small NHEJ deletions that do not allow puro^R^ gene expression. Additionally, we were unable to detect deletion events that remove more than 1881 bp of sequence because they result in deletion of either the EF1α promoter or the puro^R^ gene.

### Alu-mediated deletions between genomic Alu elements

**Fig 4:**
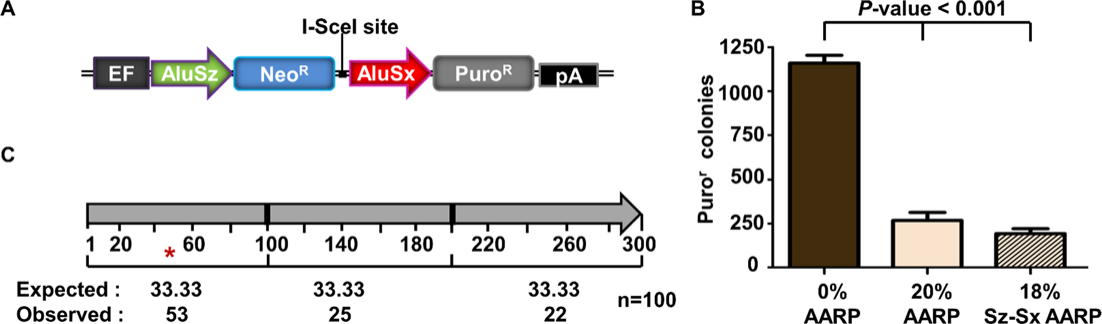
Alu/Alu recombination of disease-causing genomic Alu elements. **(A)** A schematic of the genomic AARP constructs used in this study. Two genomic Alu elements (Alu Sz and Sx) that have been shown to cause disease through Alu/Alu recombination within the MLL gene [22] were cloned into the AARP reporter cassette and integrated into HEK293FRT cells (see S1 Fig, Materials and Methods). **(B)** The average number of puro^r^ colonies is plotted for MLL-AARP (18% sequence divergence) and 20%-AARP HEK293FRT cells. Data from at least three independent experiments using at least three independently isolated clones for each AARP cell line are averaged. Error bars denote standard error and statistical significance is shown using one-way ANOVA. **(C)** Graphic representation of the location of previously reported genomic Alu/Alu recombination junctions [2,18,23–32]. The Alu/Alu recombination product is divided into three segments of equal length (100 bp). The number of Alu/Alu recombination junctions expected and observed in each 100 bp interval is shown. An asterisk (*) marks an interval in which p<0.05 significance as determined by a chi-square test for observed vs. expected.

Unlike the homeologous Alu elements tested in our AARP HEK293FRT cells described thus far, Alu elements found in the human genome do not have evenly distributed sequence divergence relative to one another. We next tested two genomic Alu elements that have been previously shown to participate in recurrent Alu-mediated deletion events in the MLL gene [22]. We cloned these Alu elements into the AARP reporter cassette to make a construct with 18% divergence between the Alu elements (Sz-Sx18%-AARP) (Fig. 4A). We tested the Alu-mediated deletion rate of Sz-Sx18%-AARP in HEK293FRT cells alongside 20%-AARP and 0%-AARP (Fig. 4B). We observed a ~4-fold decrease in both Sz-Sx18%-AARP and 20%-AARP puro^r^ colonies compared to 0%-AARP, which indicates that Alu-mediated deletions are affected by sequence divergence, but not significantly affected by the distribution of the divergence. Although the majority (88%) of repair was due to NHEJ (S10 Fig), we did observe several (12%) Alu/Alu recombination events (S11 Fig, black bars).

To determine if other genomic Alu elements behaved similarly to the MLL Alu sequences, we introduced other genomic Alu elements into the AARP reporter cassette (Sz15%-Ya5-AARP, Sx21%-Ya5-AARP, and Sz-Jb30%-AARP) and measured the rate of Alu-mediated deletions (S5 Fig). We observed an Alu-mediated deletion rate consistent with MLL Alu elements (Sz-Sx18%-AARP) and 20%-AARP HEK293FRT cells (~4-fold decrease). However, unlike with the MLL Alu elements, we did not observe any Alu/Alu recombination events between the Alu elements in Sz15%-Ya5-AARP, Sx21%-Ya5-AARP, or Sz-Jb30%-AARP HEK293FRT cells (S10 Fig). Taken together, these data indicate that sequence divergence distribution does not make a major contribution to the rate of Alu-mediated deletions.

We next asked whether the MLL Alu elements tested in our study and others [7] displayed the same recombination preference within the first 100 bp as our other diverged Alu element pairs tested. Consistent with our evenly distributed diverged Alu constructs, the MLL Alu elements (Sz-Sx18%-AARP) display a recombination hotspot in the 5’ end of the Alu element (S11 Fig). These results agree with previous analyses of 100 reported Alu/Alu recombination events (Fig. 4C) [2,18,23–32] which also show a strong bias for recombination junctions within the first 100 bp of the Alu element.

### Knockdown of Rad52

**Fig 5:**
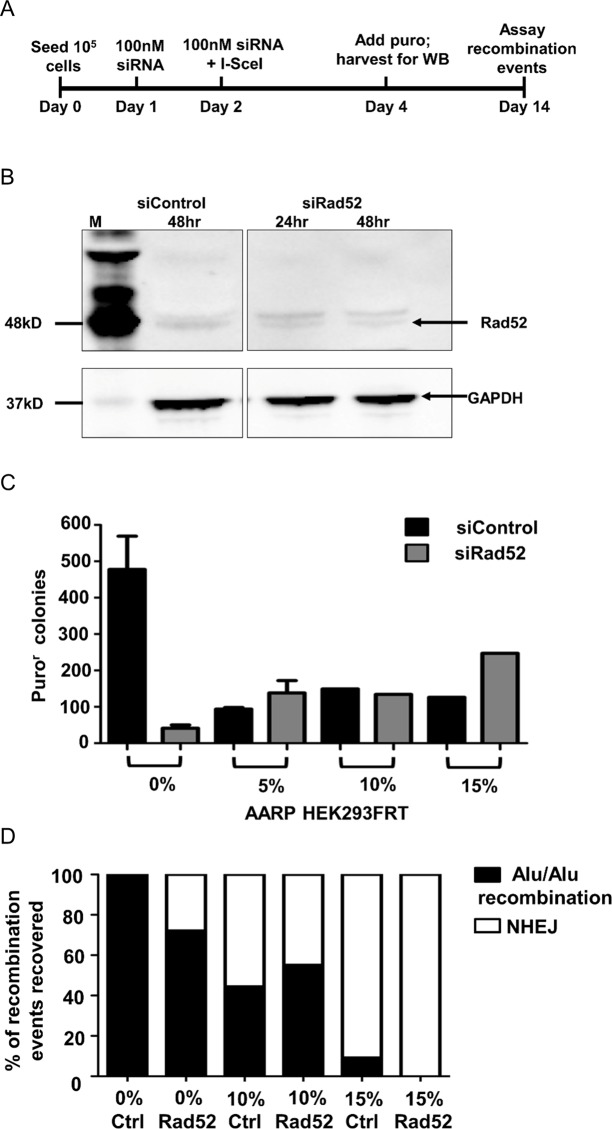
Experiments testing the effect of Rad52 siRNA knockdown in AARP HEK293FRT cells. (**A**) Experimental scheme for Rad52 knockdown experiments. Rad52 knockdown was achieved by an initial siRNA treatment followed by a subsequent siRNA treatment in conjunction with transfection of an I-SceI expression plasmid. Following treatment and I-SceI-induced DSB formation, cells were either harvested for Western blot detection of Rad52 or selected with media containing puromycin for 10 days to assay Alu-mediated deletions. (**B**) Western blot showing Rad52 siRNA knockdown compared to control siRNA treatment. M—Precision Plus Protein WesternC Standards (Bio-Rad). (**C**) The average number of puro^r^ colonies is plotted for the indicated AARP HEK293FRT cells treated with control or Rad52 siRNA. (**D**) The percentage of Alu/Alu recombination or NHEJ as determined by PCR and sequence analysis of DNA repair products from isolated puro^r^ colonies that were treated with control (Ctrl) or Rad52 siRNA is shown.

Rad52, the ssDNA binding protein involved in stabilization of DNA duplexes, is generally considered to be required for SSA repair, whereas NHEJ and MMEJ repair is Rad52-independent [9,10]. In order to confirm the role of SSA in our assays, we used siRNA against Rad52 to determine the effect of this knockdown on Alu-mediated deletions using our AARP reporter cassette in HEK293FRT cells (Fig. 5). Knockdown of Rad52 by siRNA (Fig. 5A and B) resulted in a striking decrease in Alu/Alu recombination between homologous (0%-AARP) Alu elements (Fig. 5C). PCR and sequence analysis of DNA repair events from isolated AARP HEK293FRT puro^r^ colonies from Rad52 knockdown experiments revealed that some (29%) were the result of NHEJ repair between the identical Alu elements (Fig. 5D), which is consistent with SSA being the primary mechanism of DSB resolution between homologous Alu elements. In contrast, puro^r^ colony numbers remained at similar levels in control and Rad52 siRNA treated 5%-, 10%-, and 15%-AARP HEK293FRT cells (Fig. 5C), which demonstrates that SSA is not the major pathway used for repair of homeologous Alu elements. Additionally, the similar ratio of Alu/Alu recombination to NHEJ repair for each diverged AARP reporter cassette in control and Rad52 siRNA treated cells (Fig. 5D) consistent with the ability of MMEJ repair to form ‘in register’ Alu/Alu recombination events.

### The effect of Alu element sequence divergence on DNA repair in MMR deficient cells

We hypothesized that the mismatch repair (MMR) pathway might influence the resolution of DNA DSB repair between homeologous Alu elements. In order to address the role of an essential MMR protein, Mlh1, in repair of a DSB flanked by homologous or homeologous Alu sequences, we introduced AARP reporters into the FRT site of HCT116FRT cells (denoted HCT116D cells in [33]). HCT116 cells (and the HCT116FRT derivative) are deficient for Mlh1 [34,35], a member of the MutLα heterodimer in mammalian cells that interacts with the single-base mismatch sensor, MutSα, and proliferating cell nuclear antigen (PCNA) to cause DNA nicks during the MMR process [36,37]. We observed a reduction in AARP-HCT116FRT puro^r^ colonies upon introduction of Alu element sequence divergence (S12 Fig), which indicates functional heteroduplex rejection in these cells. We saw no increase of puro^r^ colonies with AARP HCT116FRT cells 15%-30% sequence divergence range as was seen in the HEK293FRT cells (S13 Fig). These results suggest Mlh1 in HEK293FRT cells has a role in increasing the Alu-mediated deletion rate in the 15%-30% sequence divergence range, which occurs mainly through NHEJ repair.

In comparison with HEK293FRT cells, a greater proportion of repair events recovered from HCT116FRT cells were the result of Alu/Alu recombination in most diverged AARP reporters tested (S14 Fig), providing further evidence that Mlh1 contributes to variable-length NHEJ in HEK293FRT cells. We observed preferential Alu/Alu recombination within the 5’ 100 bp in HCT116FRT cells, as with HEK293FRT cells, although not to statistical significance p<0.05 (S15 Fig).

## Discussion

We have developed a novel vector system that, for the first time, allows a highly flexible measurement of intra-chromosomal Alu/Alu recombination events. Our system detects deletions caused by direct recombination between the Alu elements as well as variable-length NHEJ events. Our AAR cassette is integrated into a single genomic locus using the Flp/IN system, which allows us to measure the effect of different *cis* factors on Alu/Alu recombination in a defined chromatin environment (Fig. 1 and S1 Fig). Alu/Alu recombination events are an extremely common form of rearrangement in the human genome [6,38], which makes it important to understand whether specific traits of Alu elements contribute to their recombination potential. This is particularly relevant to specific genes in which Alu/Alu recombination events contribute a major fraction of the genetic defects observed at that locus [6,24,39].

Reports of two previous systems have measured Alu/Alu recombination directly [7,40]. The Gebow et al. reporter system relied on the presence of two identical Alu elements in two separate introns of a reporter gene. Recombination between the Alu elements removed an exon sequence resulting in a change of splicing that activated the reporter gene. This system had several drawbacks, as it utilized *hprt* as the reporter gene which did not allow easy selection in multiple cell types. The construct included both an Alu/Alu homology region for recombination and a smaller homology between the two *hprt* exon regions. Also, its dependence on random integration limited the quantitative comparison between different construct variants. A second system from the Jasin laboratory consisted of an Alu/Alu recombination assay that measured recombination between Alu elements inserted on different chromosomes using a reporter-gene approach [7]. Although this system allowed for interesting measurements, colony numbers were relatively small and it is uncertain whether interchromosomal recombination described by [7] used the same DNA repair processes as intrachromosomal recombination, the more common mechanism of Alu/Alu recombination [41,42]. In this report, we investigated for the first time the magnitude of the influence that sequence divergence between Alu elements (a major, authentic source of homology in the genome) has on DNA DSB repair. This study will allow predictions of the influence of sequence divergence on both the rate and nature DNA repair events in the vicinity of Alu elements.

**Fig 6:**
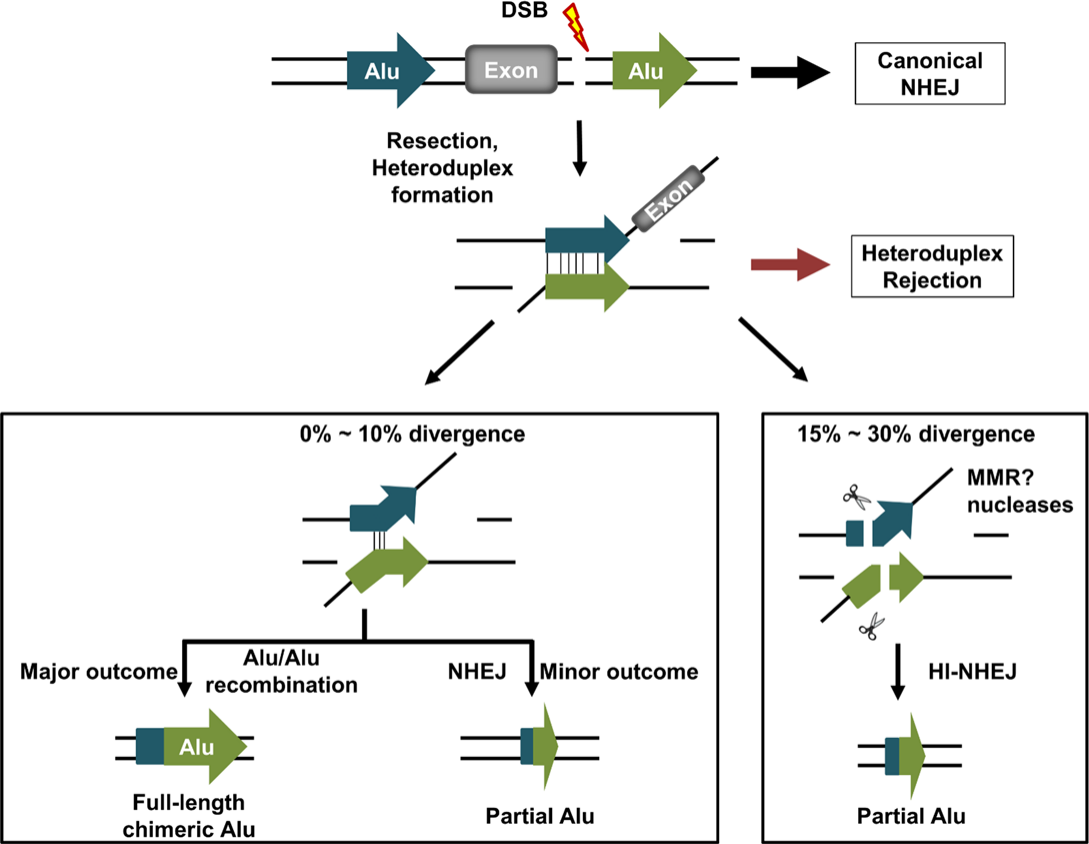
Model of DNA DSB repair between homeologous Alu elements in the human genome. A DNA double-strand break (lightning bolt) occurs between two diverged Alu elements (arrows) within the human genome. While the majority of DNA repair likely occurs through canonical NHEJ, a subset of the breaks will undergo end resection followed by Alu heteroduplex formation. Diverged Alu heteroduplexes form and then can be resolved in one of three independent ways. Slightly divergent Alu heteroduplexes (≤10%) will either undergo Alu/Alu recombination resulting in a single chimeric Alu element, or be subjected to helicase-mediated heteroduplex rejection, which will allow separated DNA ends to participate in a competing repair pathway, some of which may resolve as a chimeric or partial Alu element through MMEJ repair. We hypothesize that more divergent (15%-30%) Alu heteroduplexes may escape heteroduplex rejection and the persistent heteroduplex will be recognized and cleaved, possibly by mismatch repair (MMR), to form a new DNA end to be further processed and repaired through MMEJ, deleting some or all of the participating Alu elements (shown as partial Alu in the figure). This repair pathway we propose to call homeology-influenced NHEJ (HI-NHEJ) because it is responsive to sequence homeology (Alu elements that are 15%-30% divergent) and we hypothesize that it requires the formation of an Alu heteroduplex before repair by MMEJ.

It was not surprising to see that even low levels of sequence divergence between Alu elements (up to 10%) resulted in lower levels of Alu/Alu recombination. Presumably, this inhibitory effect is the result of heteroduplex rejection of the intermediate that forms during Alu/Alu recombination (Fig 2A and 6) [11,12,43]. Unexpectedly, highly diverged Alu substrates (15%-30%) resulted in higher levels of puromycin-activating deletions when compared to 10% Alu element sequence divergence or scrambled controls in HEK293FRT cells (Fig. 2B and S5 Fig). This observation also applied to genomic Alu elements with randomly spread divergences ranging from 15%-30% as substrates (S5 Fig). Contrary to what has been reported in plants and yeast where increasing divergence between repeat sequences results in a steady decrease in mutagenic deletions, our results indicate an enhancement of mutagenic deletions in the range of 15%-30% sequence divergence [44,45]. DNA repair junction analysis from our highly-diverged AARP HEK293FRT cells (at least 15% sequence divergence) revealed a shift away from Alu/Alu recombination towards NHEJ repair in the vicinity of the Alu elements such that 92.5% of recovered events were the result of an NHEJ deletion (S7 Fig and S8 Fig).

These data suggest that Alu elements may contribute to several competing DNA repair pathways and that the degree of Alu sequence divergence may be a critical factor in prioritizing repair pathway choice. Diverged Alu elements on either side of a DSB may still be able to form Alu heteroduplexes in the same manner as a typical SSA intermediate (Fig. 6). The recombination rate is highly sensitive to low levels of sequence divergence (≤10%), which suggests that heteroduplex rejection [11,12,43] is limiting completion of the recombination process. With greater degrees of mismatch within the Alu heteroduplex, we propose that heteroduplex rejection is less effective relative to a mismatch-related DNA cleavage event and that, rather than rejection, the Alu heteroduplexes that are formed may be preferentially subject to DNA cleavage. The likely involvement of the mismatch repair pathway is indicated by studies in *mlh1*-deficient HCT116FRT cells (Fig. 6, S12 Fig, and S13 Fig) [36,37]. We propose that this Alu heteroduplex cleavage within the Alu element is further processed to undergo a microhomology-mediated form of NHEJ (MMEJ) [7,13,46,47]. Thus, Alu elements appear to be capable of re-directing the location of NHEJ junctions in their vicinity by the formation of unstable Alu heteroduplexes in a process we call homeology-influenced NHEJ (HI-NHEJ) (Fig. 6).

HI-NHEJ potentially influences the types of genetic instability in the human genome. A number of studies have suggested the possibility that NHEJ events that lead to copy-number variation (CNV) formation may occur at a higher frequency in the vicinity of Alu elements relative to other regions [48,49]. Some studies also report an enrichment of CNV breakpoint junctions within Alu elements [50–53], which implicates Alu microhomology-mediated repair process in the formation of these CNVs [54,55].

PCR and sequence analysis of DNA repair products from AARP HEK293FRT cells with modest levels of sequence divergence (≤10%) shows 88% of the Alu/Alu recombination events contained a single-crossover region of homology within the Alu element. As others have suggested [7], these types of events are likely to be formed by MMEJ where the microhomologies are ‘in register’ between the Alu elements resulting in an Alu/Alu recombination product that is indistinguishable from an SSA product (S16A Fig). The remainder (12%) of the events did not result in a simple recombination junction, but rather represented more complex chimeras that contained patches from each Alu element (S16B Fig and S17 Fig). Formation of these complex chimeras seems inconsistent with an MMEJ repair mechanism and may reflect various resolutions of SSA repair. We believe that this is similar to the mechanism seen for resolving the heteroduplex formed by two homeologous sequences in yeast [11], where both strands are equivalent targets for mismatch repair processes, which often results in short patches of repair on both strands that sometimes create chimeras with patches of each repeat sequence. This model is consistent with our observations in Rad52 knockdown AARP HEK293FRT cells (Fig. 5). In this experiment all Alu/Alu recombination junctions characterized are the result of a single-crossover event within the Alu element (S18 Fig) rather than the formation of a complex chimeric Alu element, which suggests that DNA repair resolution as a complex chimeric Alu element is dependent on Rad52-mediated heteroduplex formation (S16B Fig and S17 Fig).

The observation that Alu/Alu recombination occurs preferentially near the 5’ region of Alu elements has been seen previously in naturally occurring examples [5,18]. Although several theories have been suggested, including a ‘chi-like’ hotspot for recombination [18] or increased accessibility to recombination proteins around the transcription factor binding sites for the RNA polymerase III promoter [6], there is no strongly supported mechanism for this preference. However, it is reassuring that our assay reproduces the types and localization patterns of Alu/Alu recombination events seen in disease-causing mutations and evolutionary CNVs. Our data do not support a previous suggestion that the preference may simply represent longer regions of perfect sequence homology between Alu elements due to some sequence conservation near the promoter.

There are several predicted consequences from our studies regarding patterns of genomic instability due to Alu elements (Fig. 6). The first is that, depending on the level of sequence divergence between any two direct repeat Alu elements in genomic proximity, competition is likely between HI-NHEJ and other DNA repair processes (SSA, ‘SSA-like events’, canonical NHEJ). At low levels of Alu element sequence divergence (≤10%), one might expect a low rate of Alu/Alu recombination and HI-NHEJ repair because these Alu heteroduplexes are likely to be successfully rejected. However, most genomic Alu elements have already accumulated relatively high levels of sequence divergence and are therefore likely to be favorable substrates for HI-NHEJ repair based on our results. Heteroduplex rejection between these divergent Alu elements may be less efficient, allowing nuclease cleavage within the Alu heteroduplex (likely by DNA mismatch repair) and further processing to undergo repair through MMEJ. Given our findings and the large contribution of Alu/Alu recombination to human genetic diseases [6], it seems likely that the actual impact of Alu elements on genetic instability, particularly through an HI-NHEJ effect, has been greatly underestimated. Our current findings provide the foundation to formulate rules for a probabilistic analysis of the genome for instability based on Alu elements.

## Materials and Methods

### Construction of Alu/Alu recombination reporter vectors and DNA manipulations

The Alu/Alu recombination puromycin (AARP) cassette was synthesized by Blue Heron Biotechnology, Inc (Bothell, WA) (http://www.blueheronbio.com) and sub-cloned in pENTR 221(Life Technologies, Grand Island, NY). The pAARP/ENTR221 construct consists of two identical Alu elements (Ya5 consensus sequence). Alu1Ya5 is flanked by *HindIII* and *BglII* restriction sites, followed by a neomycin resistance gene flanked by *NcoI* and *NdeI* restriction sites, a SV40 poly A signal and an I-SceI restriction site. The second Alu element sequence, Alu2Ya5, is flanked by *BamHI* and *EcoRI* sites and followed by a puromycin resistance gene flanked by *EcoRI* and *AgeI* sites. The Alu/Alu recombination blasticidin (AARB) cassette was constructed by insertion of a blasticidin resistance gene (codon optimized *bsr*) at the *EcoRI* and *AgeI* sites (S1B Fig). The Alu/Alu recombination eGFP (AARG) cassette was synthesized by Genescript Corporation (Piscataway, NJ), with eGFP sequence between the *EcoRI* and *AgeI* sites. pAARP/FRT_DEST, pAARB/FRT_DEST and pAARG/FRT_DEST were created in LR Clonase II plus reactions with 150 ng from each entry clone and 150 ng of pEF5/FRT/V5-DEST (Life Technologies). LR Clonase II plus reactions were performed as per the manufacturer’s protocol.

### FRT cell lines used

The following Flp-In cell lines were obtained from Life Technologies: human embryonic kidney cells (HEK 293), Baby Hamster Kidney cells (BHK), Mouse (NIH Swiss) embryonic fibroblast cells (NIH-3T3), and Chinese Hamster Ovary (CHO) cells. HCT116FRT cells were kindly provided by J. Issa (denoted HCT116D in [33]). The Flp-In site location was determined by high-throughput sequencing experiments to be located at chromosome 12:1,332,729 bp (GRCh37/hg19 assembly; S19 Fig). HEK293FRT, and BHKFRT cell lines were cultured in DMEM high glucose (Life Technologies) supplemented with 10% fetal bovine serum (Life Technologies), and NIH-3T3FRT were cultured in DMEM high glucose supplemented with 10% bovine serum (Life Technologies). CHOFRT cells were cultured in F-12 Nutrient Mixture (HAM; Life Technologies) supplemented with 10% fetal bovine serum. HCT116FRT cells were cultured in MEM supplemented with 10% fetal bovine serum, non-essential amino acids, and sodium pyruvate (Life Technologies).

### Generation of stable Alu/Alu recombination cell lines

To develop stable Alu/Alu recombination cell lines, all Alu/Alu recombination destination vectors were specifically integrated into the FRT recombination site of each parental cell line using 100 ng of destination vector and 900 ng of the FLP recombinase expression plasmid pOG44 using Lipofectamine and Plus reagent as the delivery system according to manufacturer’s protocol (Life Technologies), except for HCT116FRT cells, which used 1 μg of destination vector and 9 μg of pOG44. For these experiments, 1.0 ×10^6^ cells for HEK293FRT and HCT116FRT and 0.5 ×10^6^ cells for BHK, NIH-3T3 and CHO were seeded in a T75 culture flask 24 h prior to transfection. 48 h after transfection, medium was replaced with fresh medium containing 75 μg/ml of hygromycin for HEK293FRT cells, 150 μg/ml of hygromycin for HCT116FRT and 250 μg/ml hygromycin for all other cell lines. The medium was changed every three days. Twelve days after hygromycin selection, three independent clones were selected for Alu-mediated deletion analysis. Individual hygromycin resistant clones were expanded and maintained in medium containing 500 μg/ml neomycin (Life Technologies). As a negative control, all Alu/Alu recombination destination vectors were transfected without the pOG44 recombinase vector and no hygromycin resistant clones were detected.

### Droplet digital PCR (ddPCR) analysis of AARP copy number

Primers and probes were designed to the neomycin resistance gene of the AARP construct. The neomycin cassette probe was labeled with 5’ 6-FAM and quenched with 3’ BHQ1. Primers and probes were synthesized by IDT (Coralville, IA) (S3 Table). The RPP30 primer/probe set was purchased from Bio-Rad (Hercules, CA). Droplet digital PCR was performed using ddPCR supermix for probes (no dUTP) from Bio-Rad according to manufacturer’s protocol with 60°C annealing/extension for one minute. Droplets were read on a QX200 droplet reader and results were analyzed using QuantaSoft Analysis software (Bio-Rad).

### Generation of sequence diverged Alu/Alu recombination vectors

Sixteen sequence diverged Alu/Alu recombination constructs were generated for this study (S1 Table). All diverged Alu sequences were constructed synthetically in pUC57-simple by Blue Heron Biotechnology (Bothell, WA). Each diverged Alu1 element was isolated as a fragment obtained by *HindIII* and *BglII* digestion (New England Biolabs, Ipswich, MA), and similarly, the MLL Alu 2 (Sx) was isolated as a fragment obtained by *BamHI* and *EcoRI* digestion. These fragments then were cloned into the same sites within a linearized pAARP/ENTR221 vector and subsequently inserted into pEF5/FRT/V5-DEST using the Gateway system and recombined into cell lines using the Flp-In system as described above (Life Technologies).

### Alu/Alu recombination assays

To perform Alu-mediated deletion assays, 1.0 × 10^6^ AARP-, AARB- or AARG-HEK 293FRT, 0.5 × 10^6^ AARP-HCT116FRT, 0.5 × 10^6^ AARP-BHKFRT, 0.5 × 10^6^ AARP-NIH-3T3FRT, 0.5 × 10^6^ AARB-CHOFRT cells were seeded into T75 culture flasks one day before transfection. Cells were transfected the next day with 1.0 μg I-SceI expression vector (pCBaSce, kindly provided by M. Jasin [56]) or empty pUC19 as a control using Lipofectamine and PLUS reagent according to manufacturer protocol (Life Technologies, Grand Island, NY). For colony formation assays, puromycin (1.0 μg/ml for AARP HCT116FRT cells, 1.3 μg/ml for AARP HEK293FRT cells, and 2.0 μg/ml for all other AARP cells; Sigma-Aldrich, St. Louis, MO) or blasticidin (10 μg/ml, Invivogen, San Diego, CA) medium was added two days post-transfection and changed every three days. After 14 days, puro^r^ colonies were fixed and stained for 30 min with crystal violet (0.2% (w/v) crystal violet in 5% (v/v) acetic acid and 2.5% (v/v) isopropanol) to quantify the rate of Alu-mediated deletions. Colonies were counted using a ColCount automated colony counter from Oxford Optronix (Milton Park, Oxford, UK). Each transfection was performed a minimum of three times in parallel using independent clones in duplicates for each construct and cell type. An additional transfection was performed in parallel to isolate genomic DNA in order to analyze the DNA repair events by PCR and sequencing.

### Analysis of DNA repair events

Genomic DNA from isolated puro^r^ clones was extracted using the DNeasy Blood and Tissue kit (Qiagen, Valencia CA). 200–500 ng of gDNA was PCR amplified in a 50 μL GoTaq (Promega, Madison, WI) reaction as per manufacturer recommendations with the addition of 5% DMSO, using primers EF1-FP and Puro-RP (S2 Table). PCR products were cloned into the TOPO TA vector (TOPO TA Cloning Kit; Life Technologies) and sequenced using AARP sequencing primer (S2 Table). To test whether the distribution of the Alu/Alu recombination junctions was random, the Alu element was divided into three 100 bp segments according to the intervals of homology in which the Alu/Alu recombination junction can be mapped for each diverged AARP construct, and the observed recombination junctions were compared (using a chi-square test) to the expected number of recombination junctions based on a random distribution. Similarly, for S9A Fig and S9B Fig, the Alu element sequence was divided into 13 segments and S9C Fig into 11 segments, based on the distribution of the sequence divergence present in the different AARP reporter cassettes tested, and the statistical analysis was performed as above.

### Rad52 siRNA knockdown

Silencing of Rad52 was performed by transient gene knockdown experiments with small interfering RNAs (siRNA). Rad52 siRNA and non-specific control siRNA were obtained from Santa Cruz Biotechnology (Dallas, TX). 1x10^5^ cells were seeded at day 0 and 24 hours later, cells were treated with 100 nM siRNA duplexes. At 48 hours cells were treated with 1 μg of I-SceI expression plasmid DNA and again with 100 nM siRNA. Cells were incubated for 48 hours and re-seeded into T75 culture flasks followed by puromycin selection. All siRNA treatments and DNA-siRNA co-treatments were done using Oligofectamine reagent (Invitrogen) as per manufacturer recommendations.

### Rad52 western blot

Protein was extracted from cell lysates using TLB-SDS buffer (0.5% (w/v) SDS, 0.5% (v/v) Triton X-100, 150 mM NaCl, 10 mM EDTA, 50 mM Tris pH 7.2). Protein was separated on a 4–12% Tris-Glycine gel (Life Technologies) and blotted using a mouse monoclonal antibody specific to Rad52 (Santa Cruz Biotechnology). Rad52 detection was done using a goat anti-mouse HRP conjugated antibody (Santa Cruz Biotechnology) and visualized and quantified on a ChemiDoc (BioRad).

### Flow cytometry

AARG-HEK293FRT cells were collected 72 hours after induction of DSB by I-SceI and eGFP fluorescence was analyzed on an LSR II flow cytometer (BD Biosciences, Franklin Lakes, NJ).

### Statistical analysis

For most experiments, statistical evaluation was performed by two-tailed, Student’s t-tests using GraphPadPrism 5 software. For statistical analysis between 0%-AARP and diverged AARP constructs, (Fig. 2B), one-way ANOVA was used using GraphPad Prism 5. Error bars represent the standard error of the mean (SEM) of at least 3 independent experiments. *P* values were considered significant if p <0.05.

## Supporting Information

S1 Fig Generation of Flp-In AARP stable cell lines. (**A**) Diagram of the pFRT/*lac*Zeo Flp-In target site vector integrated in the parental cell lines containing FRT sites (Life Technologies). Cells with integrated pFRT/*lac*Zeo were zeocin resistant (zeo^r^). (**B**) The AARP stable cell lines were created by site-specific recombination at the FRT site. The AARP reporter cassette contains a hygromycin resistance (hygro^R^) gene used to select a stable cell line upon Flp-recombinase-mediated integration of the cassette. The AARP reporter cassette contains a human elongation factor 1α (EF1α) promoter upstream of a neomycin resistance (neo^R^) gene. An I-SceI endonuclease cleavage site is positioned between the two Alu elements. Prior to DNA repair, the puromycin resistance (puro^R^) gene was not expressed due to distance from the EF1α promoter and interruption by the neo^R^ gene and ployadenylation (pA) site, which results in puromycin sensitivity (puro^s^). (**C**) Repair of I-SceI-induced DNA double-strand breaks (DSBs) through Alu/Alu recombination or NHEJ that deletes a sufficient portion of sequence allowed puro^R^ gene expression and selection of repaired AARP cells in media containing puromycin.
(TIF)

S2 Fig Droplet digital PCR determination of AARP copy number in HEK293FRT cells. (**A**) Schematic of the ddPCR assay. A droplet digital PCR (ddPCR) assay was used to confirm that the AARP cassette was integrated as a single copy into HEK293FRT cells. Two Taqman style primer/probe sets were used (S3 Table). The first set recognized the neo^R^ gene integrated as part of the AARP reporter using a 5’ 6-FAM-labelled probe (6-FAM moiety indicated by the blue circle). The second primer/probe set used a HEX-labelled RPP30 probe (HEX moiety indicated by the green circle), which detected a cellular housekeeping gene, RPP30, present as diploid in HEK293FRT cells. (**B**) Results of the ddPCR assay with parental HEK293FRT cell genomic DNA. During ddPCR, genomic DNA for each sample was partitioned into droplets, which are then thermocycled to the plateau phase of PCR. Each droplet was then measured for FAM and HEX fluorescence and plotted accordingly (FAM; neo^R^ gene, y-axis) (HEX; RPP30 gene, x-axis). Droplets with HEX fluorescence (RPP30 gene, green cluster and orange dots) were readily detected from ddPCR with parental HEK293FRT cell genomic DNA, whereas very few droplets displayed FAM fluorescence (neo^R^ gene, blue and orange dots). These FAM-positive droplets were likely due to non-specific amplification of genomic DNA in these droplets. (**C**) Results of the ddPCR assay with AARP HEK293FRT cell genomic DNA. Droplets were measured and plotted as in (**B**). In ddPCR assays with AARP HEK293FRT cell genomic DNA both HEX (RPP30 gene) and FAM (neo^R^ gene) fluorescence was detected. (**D**) The concentration of the neo^R^ and RPP30 gene DNA in samples tested. The concentration of the neo^R^ and RPP30 gene DNA is shown as calcutated by the QuantaSoft ddPCR software (Bio-Rad) as copies/μL of input genomic DNA from parental HEK293FRT cells and five different clones of AARP HEK293FRT cells, showing the RPP30 gene exists at roughly twice the copy number of the neo^R^ gene. (**E**) The copy number of the neo^r^ gene relative to the RPP30 gene in samples tested. The relative copy number of the neo^R^ gene with respect to the RPP30 gene is plotted for genomic DNA tested in (**D**). These results confirm that the neo^R^ gene exists at half the copy number of the RPP30 gene in various AARP HEK293FRT clones.
(TIF)

S3 Fig The Alu/Alu recombination reporter can be used in multiple cell lines. The AARP cassette was stably integrated into (**A**) baby hamster kidney (BHK) FRT cells and (**B**) mouse embryonic fibroblast (NIH-3T3) FRT and the AARB reporter was stably integrated into (**C**) Chinese hamster ovary (CHO) FRT cells (Materials and Methods). Each cell line was transfected with either an I-SceI endonuclease vector to induce DNA DSBs or empty vector (puc19) as a negative control. The average numbers of puro^r^ or blast^r^ colonies are plotted as a function of I-SceI endonuclease or pUC19 vector treatment. Data from three independently isolated clones for each cell line are averaged with error bars indicating standard error of the mean. Statistical significance is shown using one-way ANOVA and p-values for each cell line are indicated. Representative images of T75 culture flasks with stained puro^r^ or blast^r^ colonies for each of the three cell lines are shown below graphs.
(TIF)

S4 Fig Sequence alignments of the Alu elements used in the AARP reporter cassettes are shown. The 0% Alu (Alu Ya5 consensus sequence) is the sequence of the Alu elements used in the 0%-AARP cassette. In each of the diverged AARP reporter cassettes, Alu 1 was replaced with a diverged Alu element by subcloning. The sequence alignments of each diverged Alu sequence used is shown with diverged nucleotides relative to 0% Alu marked at the respective positions in red.
(TIF)

S5 Fig Alu/Alu recombination of genomic Alu elements. Three genomic Alu element pairs (Sz15%-Ya5-AARP, Sx21%-Ya5-AARP, and Sz-Jb30%-AARP) as well as two scrambled-sequence Alu element pairs (Scr1- and Scr2-AARP, 75%) were introduced into AARP and integrated into HEK293FRT cells (see S1 Fig, Materials and Methods). The average number of puro^r^ colonies is plotted for these cells, along with 15%-, 20%-, 30%-AARP HEK293FRT cells. Data from at least three independently isolated clones for each AARP cell line are averaged with error bars indicating standard error.
(TIF)

S6 Fig An image of a 1% TAE agarose gel of DNA repair products from eight isolated puro^r^ 3%-AARP HEK293FRT cells following PCR amplification is shown. The size of the Alu/Alu recombination product is ~1.5 kb (lanes 1, 2, 4, 5, 6, and 8) while NHEJ repair products have variable sizes (lanes 3 and 7). M = 1 kb DNA ladder (Promega).
(TIF)

S7 Fig NHEJ repair junctions from isolated puro^r^ AARP HEK293FRT colonies following I-SceI expression vector transfection. At the top is a schematic of the NHEJ product from our AARP vector system where the Neo^R^ gene has been deleted along with portions of the Alu elements on both sides. The NHEJ repair junctions recovered from isolated puro^r^ colonies in the diverged AARP constructs indicated are shown as the size of the variable deletions along with the sequences flanking the deletion on both sides. Deletions ranged in size from 897 base pairs to 1901 base pairs. Alu element sequences are in bold italics. Alu1 or flanking AARP reporter cassette sequence is shown to the left of the NHEJ deletion and Alu2 or flanking AARP reporter cassette sequence is shown to the right of the NHEJ deletion. Underlined sequences indicate a microhomology found at the NHEJ repair junction. The same sequences are shown more schematically in S8 Fig.
(TIF)

S8 Fig Schematic representation of the NHEJ deletions from AARP HEK293FRT cells. A schematic representation of the data presented in S7 Fig shows the location of the NHEJ deletion events observed in the diverged AARP HEK293FRT cells indicated. Horizontal red bars represent the region of the AARP reporter cassette deleted in each of the isolated puro^r^ AARP HEK293FRT colonies tested.
(TIF)

S9 Fig Addition of sequence homology does not alter the prevalence of Alu/Alu recombination junctions within the 5’ end of the Alu element. The Alu1 sequence in 5%-AARP was modified to include larger stretches of homology to Alu2 (Alu Ya5 consensus sequence) and subsequently inserted into HEK293FRT cells (see S1 Fig, Materials and Methods). (**A**) The distribution of Alu/Alu recombination junctions for 5%e-AARP (40 bp stretches of sequence homology at the 5’ and 3’ ends of Alu1) HEK293FRT cells was determined by PCR and sequence analysis of DNA repair products from isolated puro^r^ colonies. The Alu/Alu recombination product is divided into segments according to the intervals of homology in which the Alu/Alu recombination junction can be mapped. The number of Alu/Alu recombination junctions expected and observed in each interval is shown. While not statistically significant at p<0.05, there is still a slight preference for resolution of repair at the 5’ end of the Alu element. (**B**) The distribution of Alu/Alu recombination junctions for 5%2M-AARP (contains two stretches of 40 bp of sequence homology in the middle of Alu1) HEK293FRT cells was determined as described in (**A**). An asterisk (*) marks an interval in which p<0.05 significance as determined by a chi-square test for observed vs. expected. No increase in recombination within the stretches of homology was observed. In fact, we again observe a statistically significant bias for recombination resolution within the first 100 base pairs of the Alu element. (**C**) The distribution of Alu/Alu recombination junctions for 5%4M-AARP (contains 100 bp of sequence homology in the middle of Alu1) HEK293FRT cells was determined as described in (**A**). An asterisk (*) marks an interval in which p<0.05 significance as determined by a chi-square test for observed vs. expected. No significant increase in recombination within the stretch of homology was observed. Again, however, we observed a statistically significant increase in recombination repair junctions within the first 100 base pairs of the Alu element compared to the number expected based on a random distribution.
(TIF)

S10 Fig NHEJ repair junctions from Sz15%-Ya5-, Sx21%-Ya5-, and Sz-Jb30%-AARP HEK293FRT cells (Fig 4 and S5 Fig) is shown as in Fig. 7. The NHEJ repair junctions recovered from isolated puror colonies are shown as the size of the variable deletions along with the sequences flanking the deletion on both sides. Deletions ranged in size from 897 base pairs to 1901 base pairs. Alu element sequences are in bold italics. Alu1 or flanking AARP reporter cassette sequence is shown to the left of the NHEJ deletion and Alu2 or flanking AARP reporter cassette sequence is shown to the right of the NHEJ deletion. Underlined sequences indicate a microhomology found at the NHEJ repair junction.
(TIF)

S11 Fig The sequence alignment of the MLL Alu Sz and Sx tested in Fig. 4 shows the location of the Alu/Alu recombination junctions reported in this study and previous studies. Black bars above the sequence represent the interval containing the Alu/Alu recombination junctions reported in this study. Similarly, blue bars represent those reported by [7] and orange bars those reported by [23,24].
(TIF)

S12 Fig The effect of sequence divergence on Alu/Alu recombination in HCT116FRT cells. Stably integrated AARP HCT116 cell lines were generated using the Flp-In system (see S1 Fig, Materials and Methods) and transfected with I-SceI expression vector to induce DNA DSBs. The average number of puro^r^ colonies is plotted for the indicated AARP HCT116FRT cells tested. Data from at least three independent experiments using at least three independently isolated clones for each AARP cell line are averaged with error bars indicating standard error.
(TIF)

S13 Fig The effect of sequence divergence on Alu/Alu recombination in HCT116FRT cells relative to HEK293FRT cells. The average number of puro^r^ colonies for AARP HEK293FRT cells (Fig. 2B and Table 1) and AARP HCT116FRT cells (S12 Fig) is shown with 0%-AARP colony numbers normalized to 100. Data from at least three independent experiments using at least three independently isolated clones for each AARP cell line are averaged with error bars indicating standard error. AARP HCT116FRT cells lack the enhancement of puro^r^ colony formation within the 15–30% Alu element sequence divergence as observed with HEK293FRT cells.
(TIF)

S14 Fig The effect of sequence divergence on DNA repair pathway usage in AARP HEK293FRT and AARP HCT116FRT cells. The percentage of Alu/Alu recombination or NHEJ as determined by PCR and sequence analysis of DNA repair products from isolated puro^r^ colonies is shown.
(TIF)

S15 Fig The distribution of Alu/Alu recombination junctions in AARP HCT116FRT cells. **(A)** A schematic of the Alu/Alu recombination product as shown in Fig. 3A. **(B)** The distribution of Alu/Alu recombination junctions in diverged AARP HCT116FRT cells as determined by PCR and sequence analysis of DNA repair products from isolated puro^r^ colonies. The Alu/Alu recombination product is divided into three segments of equal length (100 bp), which each contain the same extent of sequence divergence. The number of Alu/Alu recombination junctions expected and observed in each 100 bp interval is shown.
(TIF)

S16 Fig Schematic of Alu chimeras obtained from diverged AARP HEK293FRT cells. **(A)** Schematic showing the typical Alu/Alu recombination product observed. The majority of intact Alu elements show a single conversion location within the Alu element, which implicates MMEJ as the repair pathway used for the majority of Alu/Alu recombination events between diverged Alu elements. **(B)** Schematics showing the complex Alu element complex chimeras generated in a minority of Alu/Alu recombination events in 5%-, 10%-, and 15%-AARP HEK293FRT cells. Multiple conversion locations can be observed in these Alu/Alu recombination events, which implicates repair of mismatched tracts within a SSA heteroduplex intermediate to generate “patches” of Alu1 and Alu2 in each AARP clone characterized.
(TIF)

S17 Fig Sequences of Alu complex chimaeras obtained from diverged AARP HEK293FRT cells. Sequence alignments of the complex chimeras generated during Alu/Alu recombination events described schematically in S17 Fig are shown for **(A)** 5%-AARP, **(B)** 10%-AARP, and **(C)** 15%-AARP HEK293FRT cells.
(TIF)

S18 Fig The distribution of Alu/Alu recombination junctions in control and Rad52 siRNA-treated AARP HEK293FRT cells. **(A)** A schematic of the Alu/Alu recombination product as shown in Fig. 3A. **(B)** The distribution of Alu/Alu recombination junctions in indicated AARP HEK293FRT cells treated with Rad52 siRNAs as determined by PCR and sequence analysis of DNA repair products from isolated puro^r^ colonies. The Alu/Alu recombination product is divided into three segments of equal length (100 bp), which each contain the same extent of sequence divergence. The number of Alu/Alu recombination junctions expected and observed in each 100 bp interval is shown. An asterisk (*) marks an interval in which p<0.05 significance as determined by a chi-square test for observed vs. expected.
(TIF)

S19 Fig The genomic location of the Flp-In site in HEK293FRT cells. Both sequence junctions of the integration of the pFRT/*lac*Zeo vector at Chr 12 in HEK293FRT cells (HEK293-based Flp-In cells; Life Technologies) are shown as determined by whole genome sequencing with Illumina Next Generation Sequencing.
(TIF)

S1 Table The names of the variant versions of the AARP vector shown in more detail in S1 Fig are shown. The names incorporate the percent divergence between Alu2 (Ya5 consensus) and the variant Alu1 placed in most of the vectors. Most of the actual Alu1 sequences for these vectors are shown in S4 Fig. The 5%e represents evenly spaced mutations every 20 bp but with the ends having twice the length of consensus sequence match. The 5%2M and 5%4M in names refers to a stretch of either 80 or 100 bp of consensus sequence match, respectively, located in the center of the Alu. The locations of these longer homology stretches are shown schematically in S9 Fig. The Scr1- and Scr2-AARP names refer to two versions of totally randomized sequence replacing Alu1 (75%). The Sz, Sx and Jb nomenclature refers to natural Alu elements that are described in the Results.
(DOCX)

S2 Table The oligonucleotides used for PCR amplification and sequencing across the DNA repair junctions are shown. (DOCX)

S3 Table The oligonucleotides used in droplet digital PCR for copy number validation are shown. The results of this experiment are shown in S2 Fig.
(DOCX)

S4 Table The percent sequence divergence of the Alu elements used in AARP HEK293FRT cells is listed and corresponds to the sequences in S4 Fig. In each experiment 10^6^ cells are transfected with control (No DSBs) or I-SceI expression plasmid. Because no replating occurs before colony formation, the number of repair events measured by puro^r^ colonies directly correlates with the repair frequency and it has been adjusted to frequency per 100,000 cells for more direct comparison with other studies and given with standard error.
(DOCX)
