# Supplementary figures and images for: The Contribution of Alu Elements to Mutagenic DNA Double-Strand Break Repair
Source: PLoS Genet. 2015 Mar 11;11(3):e1005016. doi: 10.1371/journal.pgen.1005016 (PMC4356517; doi:10.1371/journal.pgen.1005016)

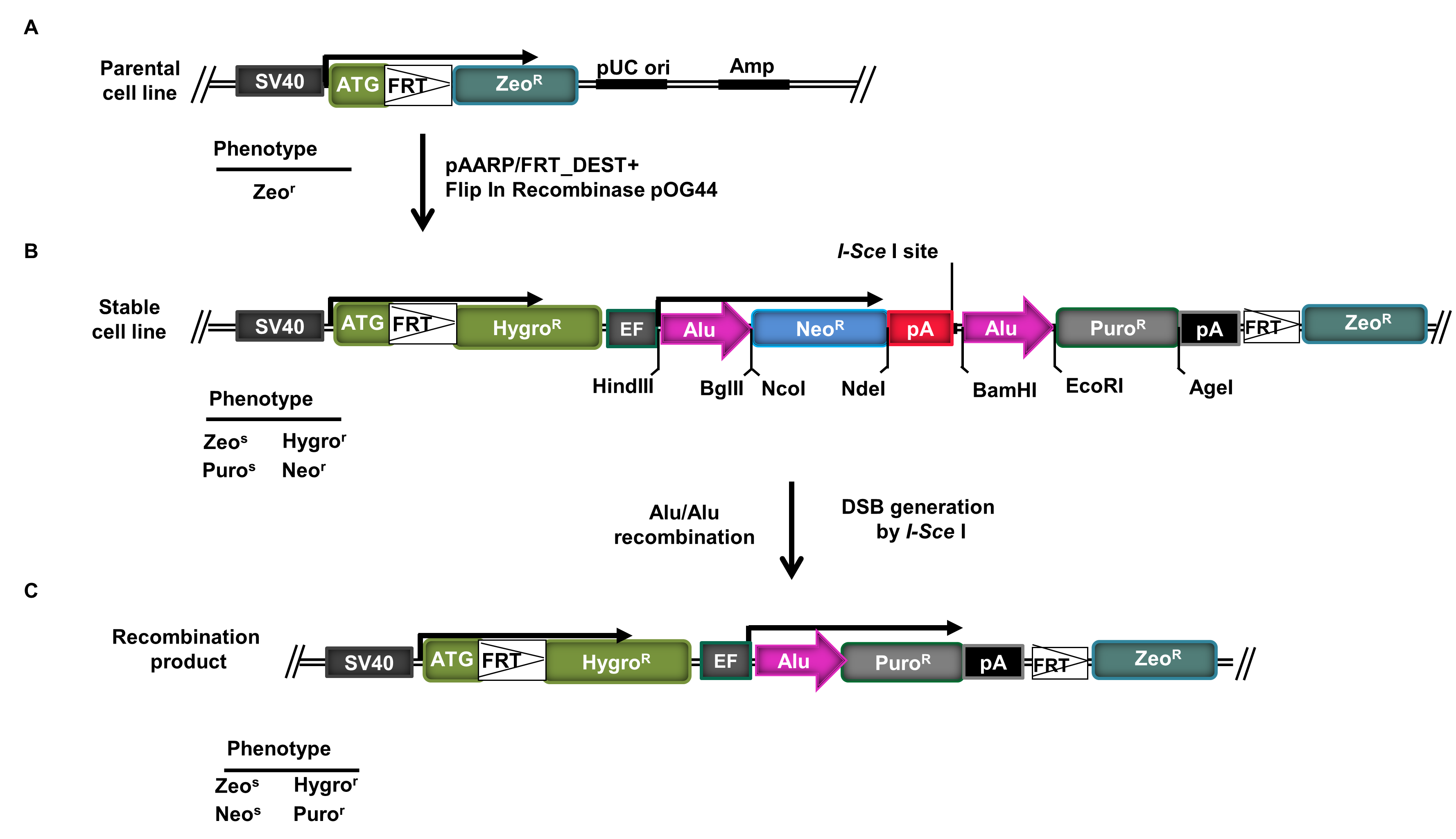

Supplement: S1 Fig — (A) Diagram of the pFRT/lacZeo Flp-In target site vector integrated in the parental cell lines containing FRT sites (Life Technologies). Cells with integrated pFRT/lacZeo were zeocin resistant (zeor). (B) The AARP stable cell lines were created by site-specific recombination at the FRT site. The AARP reporter cassette contains a hygromycin resistance (hygroR) gene used to select a stable cell line upon Flp-recombinase-mediated integration of the cassette. The AARP reporter cassette contains a human elongation factor 1α (EF1α) promoter upstream of a neomycin resistance (neoR) gene. An I-SceI endonuclease cleavage site is positioned between the two Alu elements. Prior to DNA repair, the puromycin resistance (puroR) gene was not expressed due to distance from the EF1α promoter and interruption by the neoR gene and ployadenylation (pA) site, which results in puromycin sensitivity (puros). (C) Repair of I-SceI-induced DNA double-strand breaks (DSBs) through Alu/Alu recombination or NHEJ that deletes a sufficient portion of sequence allowed puroR gene expression and selection of repaired AARP cells in media containing puromycin. (TIF) [file pgen.1005016.s001.tif]

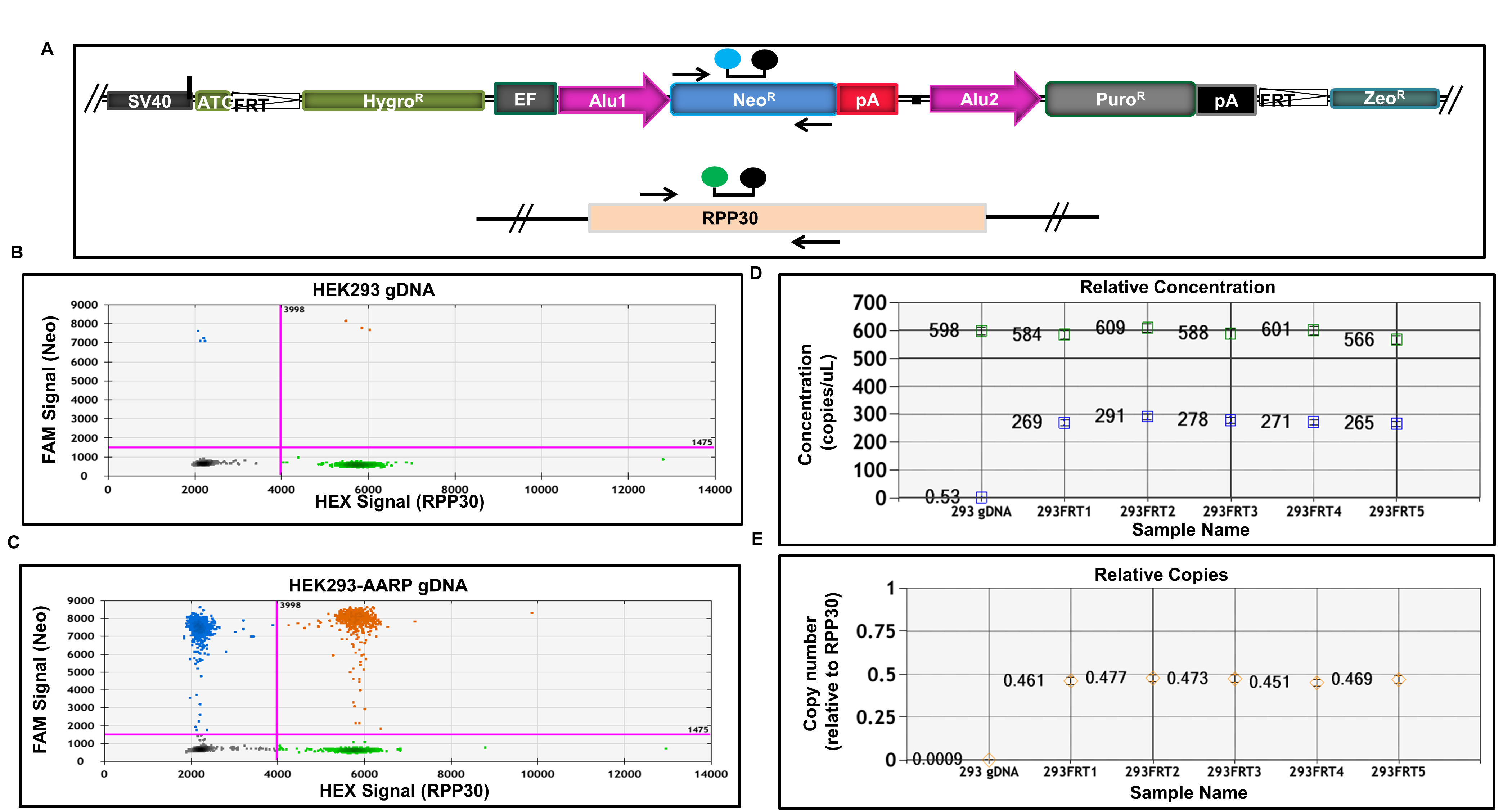

Supplement: S2 Fig — (A) Schematic of the ddPCR assay. A droplet digital PCR (ddPCR) assay was used to confirm that the AARP cassette was integrated as a single copy into HEK293FRT cells. Two Taqman style primer/probe sets were used (S3 Table). The first set recognized the neoR gene integrated as part of the AARP reporter using a 5’ 6-FAM-labelled probe (6-FAM moiety indicated by the blue circle). The second primer/probe set used a HEX-labelled RPP30 probe (HEX moiety indicated by the green circle), which detected a cellular housekeeping gene, RPP30, present as diploid in HEK293FRT cells. (B) Results of the ddPCR assay with parental HEK293FRT cell genomic DNA. During ddPCR, genomic DNA for each sample was partitioned into droplets, which are then thermocycled to the plateau phase of PCR. Each droplet was then measured for FAM and HEX fluorescence and plotted accordingly (FAM; neoR gene, y-axis) (HEX; RPP30 gene, x-axis). Droplets with HEX fluorescence (RPP30 gene, green cluster and orange dots) were readily detected from ddPCR with parental HEK293FRT cell genomic DNA, whereas very few droplets displayed FAM fluorescence (neoR gene, blue and orange dots). These FAM-positive droplets were likely due to non-specific amplification of genomic DNA in these droplets. (C) Results of the ddPCR assay with AARP HEK293FRT cell genomic DNA. Droplets were measured and plotted as in (B). In ddPCR assays with AARP HEK293FRT cell genomic DNA both HEX (RPP30 gene) and FAM (neoR gene) fluorescence was detected. (D) The concentration of the neoR and RPP30 gene DNA in samples tested. The concentration of the neoR and RPP30 gene DNA is shown as calcutated by the QuantaSoft ddPCR software (Bio-Rad) as copies/μL of input genomic DNA from parental HEK293FRT cells and five different clones of AARP HEK293FRT cells, showing the RPP30 gene exists at roughly twice the copy number of the neoR gene. (E) The copy number of the neor gene relative to the RPP30 gene in samples tested. The relative copy number of the neoR [file pgen.1005016.s002.tif]

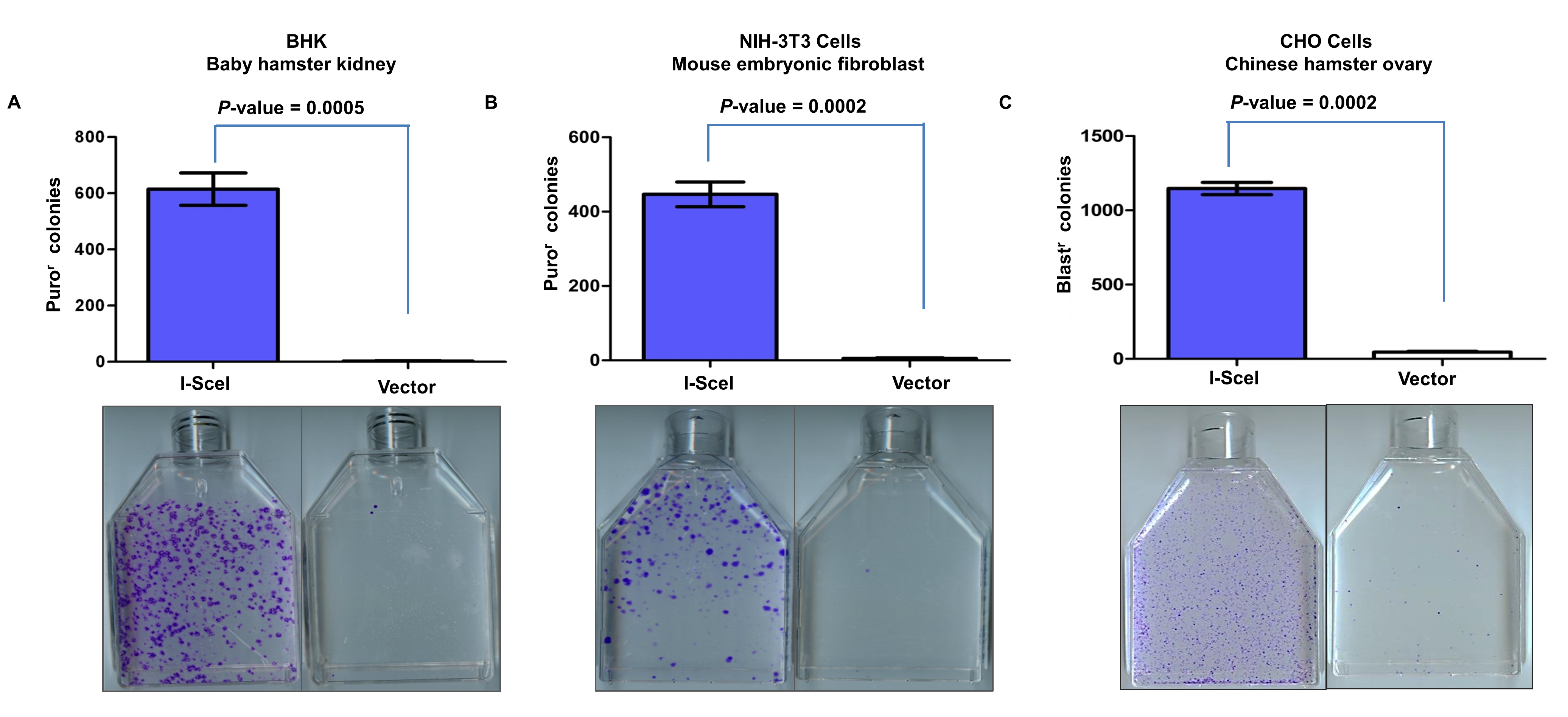

Supplement: S3 Fig — The AARP cassette was stably integrated into (A) baby hamster kidney (BHK) FRT cells and (B) mouse embryonic fibroblast (NIH-3T3) FRT and the AARB reporter was stably integrated into (C) Chinese hamster ovary (CHO) FRT cells (Materials and Methods). Each cell line was transfected with either an I-SceI endonuclease vector to induce DNA DSBs or empty vector (puc19) as a negative control. The average numbers of puror or blastr colonies are plotted as a function of I-SceI endonuclease or pUC19 vector treatment. Data from three independently isolated clones for each cell line are averaged with error bars indicating standard error of the mean. Statistical significance is shown using one-way ANOVA and p-values for each cell line are indicated. Representative images of T75 culture flasks with stained puror or blastr colonies for each of the three cell lines are shown below graphs. (TIF) [file pgen.1005016.s003.tif]

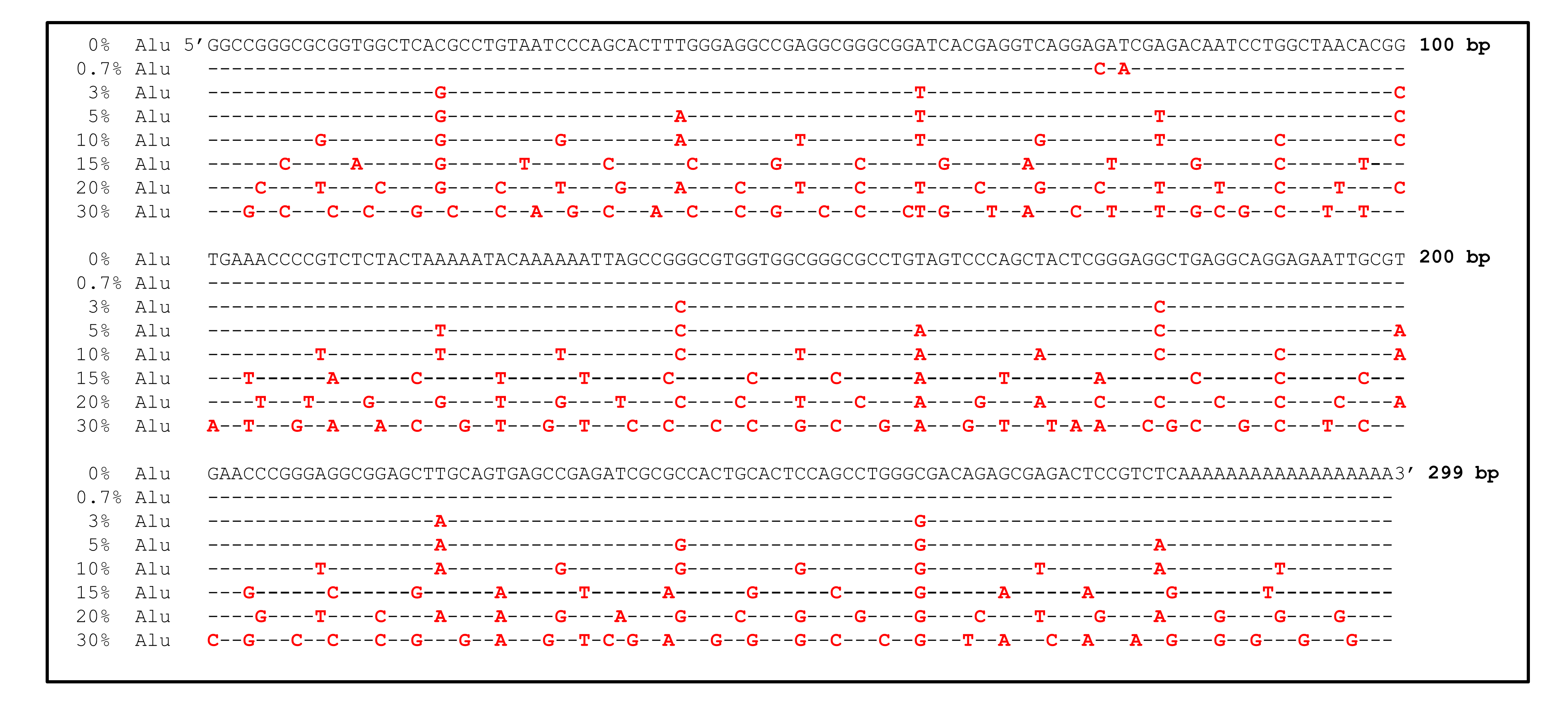

Supplement: S4 Fig — The 0% Alu (Alu Ya5 consensus sequence) is the sequence of the Alu elements used in the 0%-AARP cassette. In each of the diverged AARP reporter cassettes, Alu 1 was replaced with a diverged Alu element by subcloning. The sequence alignments of each diverged Alu sequence used is shown with diverged nucleotides relative to 0% Alu marked at the respective positions in red. (TIF) [file pgen.1005016.s004.tif]

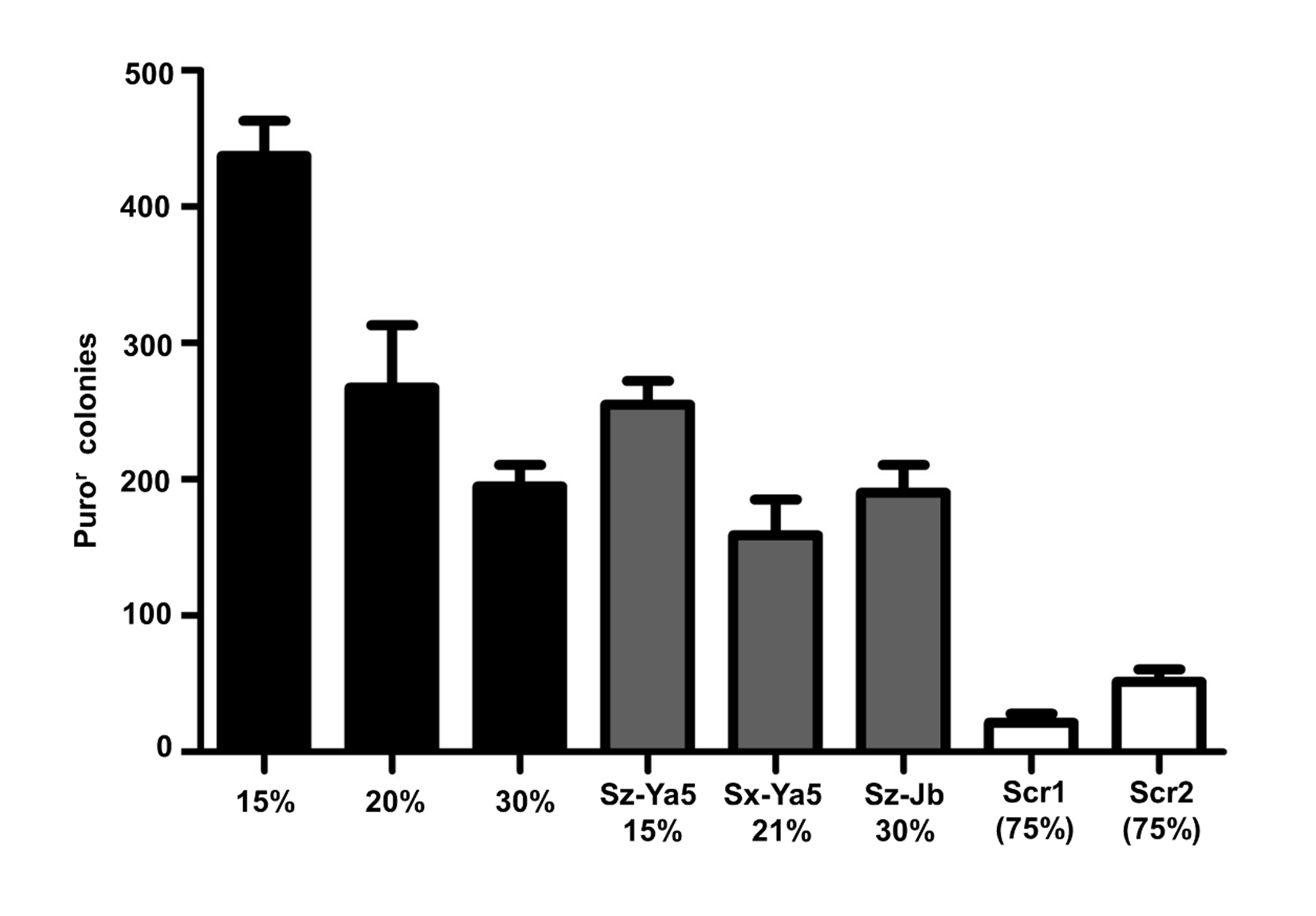

Supplement: S5 Fig — Three genomic Alu element pairs (Sz15%-Ya5-AARP, Sx21%-Ya5-AARP, and Sz-Jb30%-AARP) as well as two scrambled-sequence Alu element pairs (Scr1- and Scr2-AARP, 75%) were introduced into AARP and integrated into HEK293FRT cells (see S1 Fig, Materials and Methods). The average number of puror colonies is plotted for these cells, along with 15%-, 20%-, 30%-AARP HEK293FRT cells. Data from at least three independently isolated clones for each AARP cell line are averaged with error bars indicating standard error. (TIF) [file pgen.1005016.s005.tif]

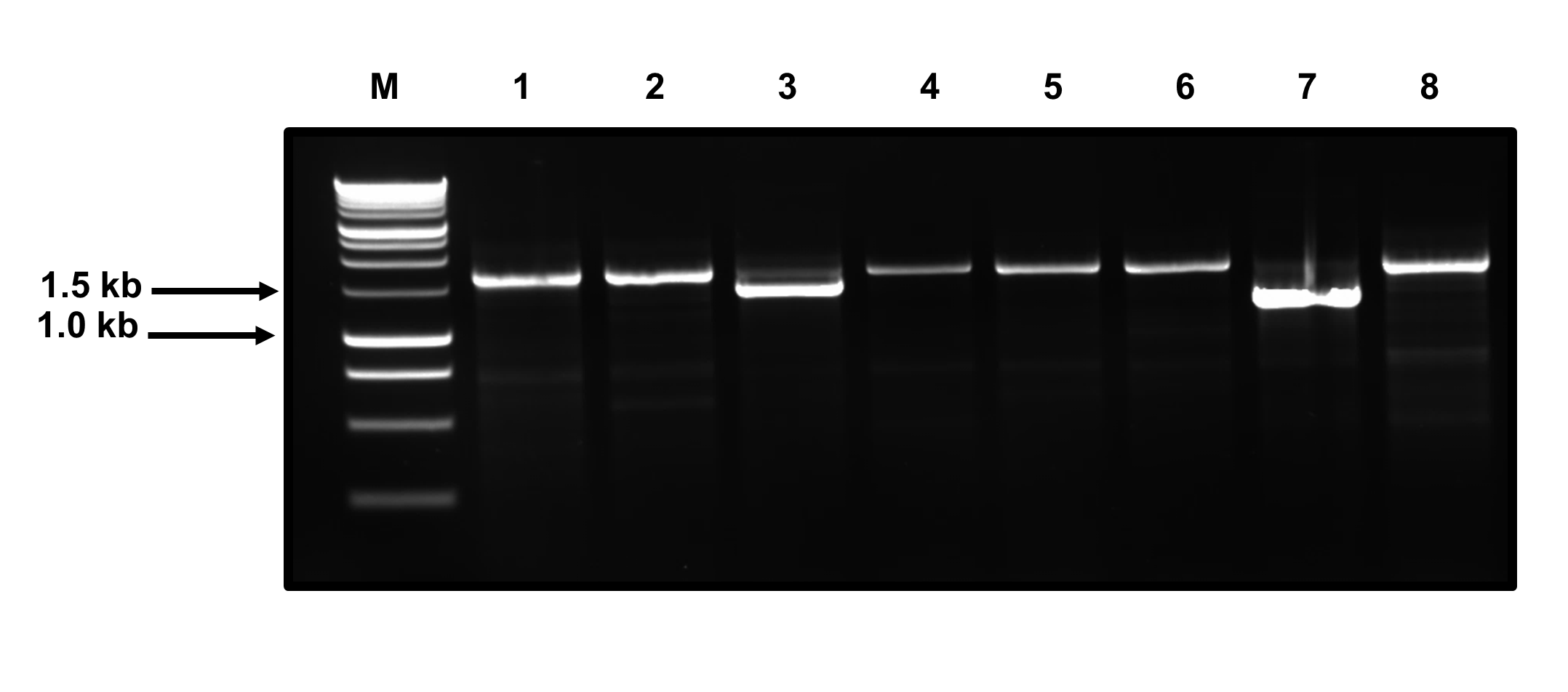

Supplement: S6 Fig — The size of the Alu/Alu recombination product is ~1.5 kb (lanes 1, 2, 4, 5, 6, and 8) while NHEJ repair products have variable sizes (lanes 3 and 7). M = 1 kb DNA ladder (Promega). (TIF) [file pgen.1005016.s006.tif]

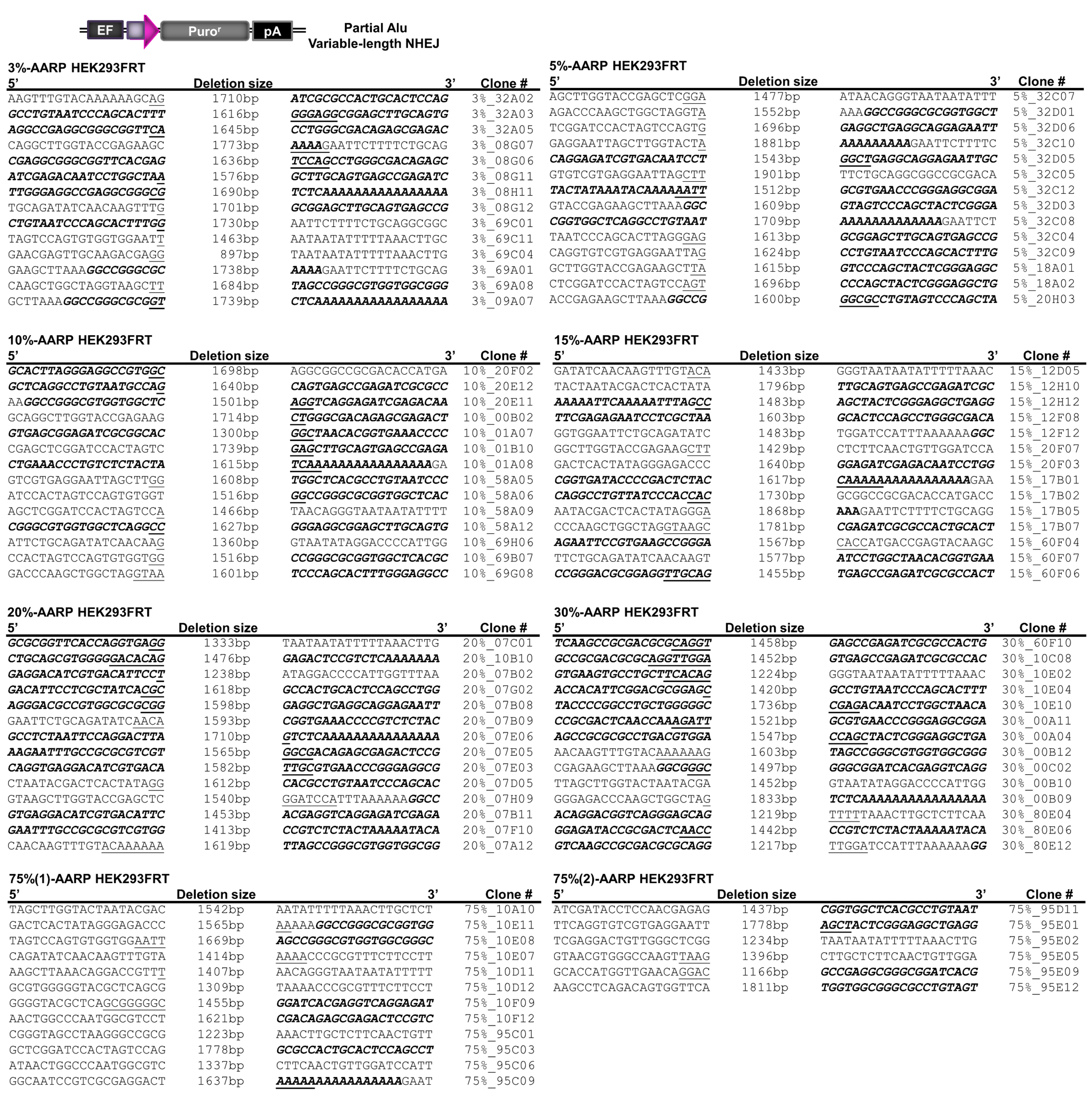

Supplement: S7 Fig — At the top is a schematic of the NHEJ product from our AARP vector system where the NeoR gene has been deleted along with portions of the Alu elements on both sides. The NHEJ repair junctions recovered from isolated puror colonies in the diverged AARP constructs indicated are shown as the size of the variable deletions along with the sequences flanking the deletion on both sides. Deletions ranged in size from 897 base pairs to 1901 base pairs. Alu element sequences are in bold italics. Alu1 or flanking AARP reporter cassette sequence is shown to the left of the NHEJ deletion and Alu2 or flanking AARP reporter cassette sequence is shown to the right of the NHEJ deletion. Underlined sequences indicate a microhomology found at the NHEJ repair junction. The same sequences are shown more schematically in S8 Fig. (TIF) [file pgen.1005016.s007.tif]

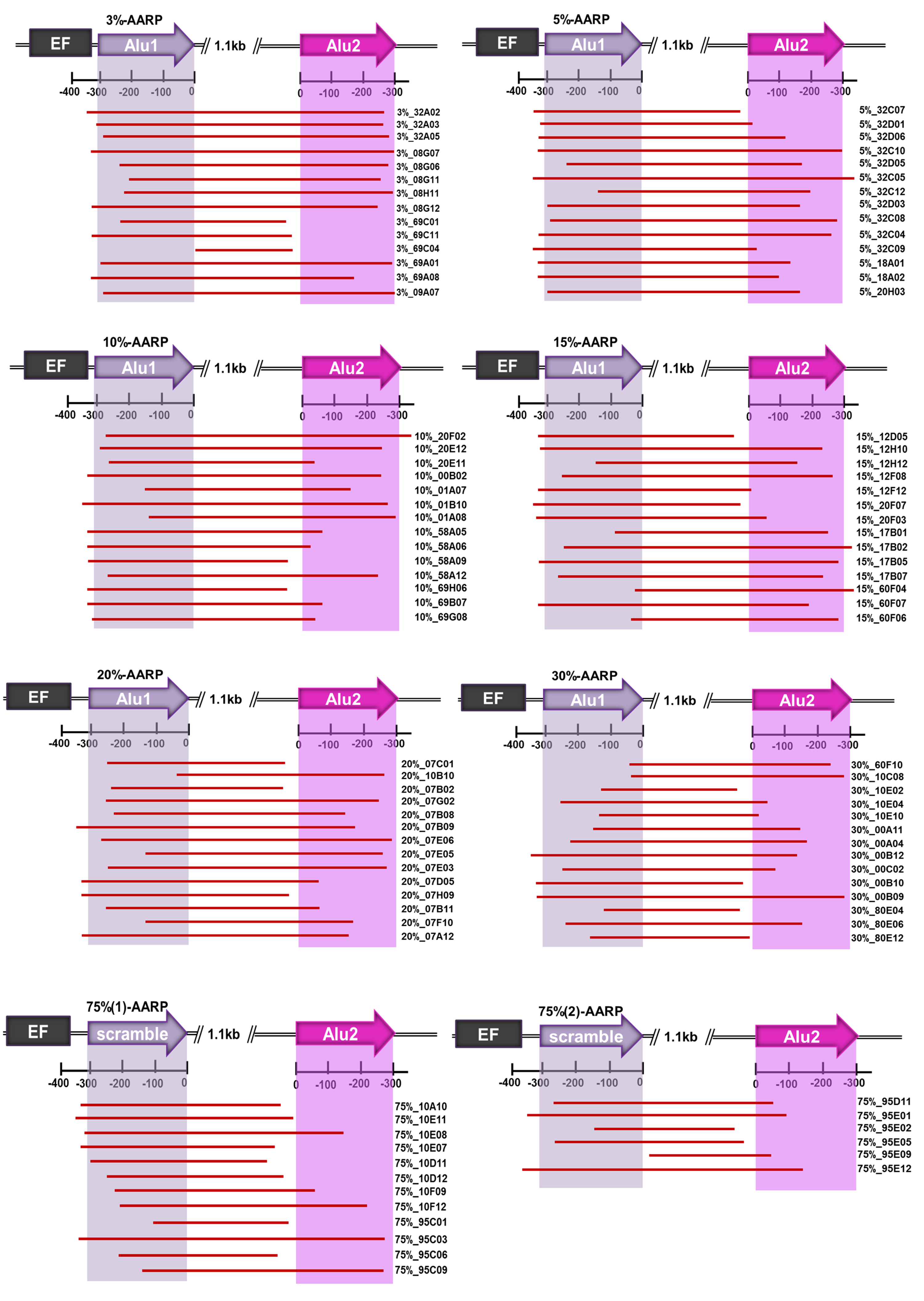

Supplement: S8 Fig — A schematic representation of the data presented in S7 Fig shows the location of the NHEJ deletion events observed in the diverged AARP HEK293FRT cells indicated. Horizontal red bars represent the region of the AARP reporter cassette deleted in each of the isolated puror AARP HEK293FRT colonies tested. (TIF) [file pgen.1005016.s008.tif]

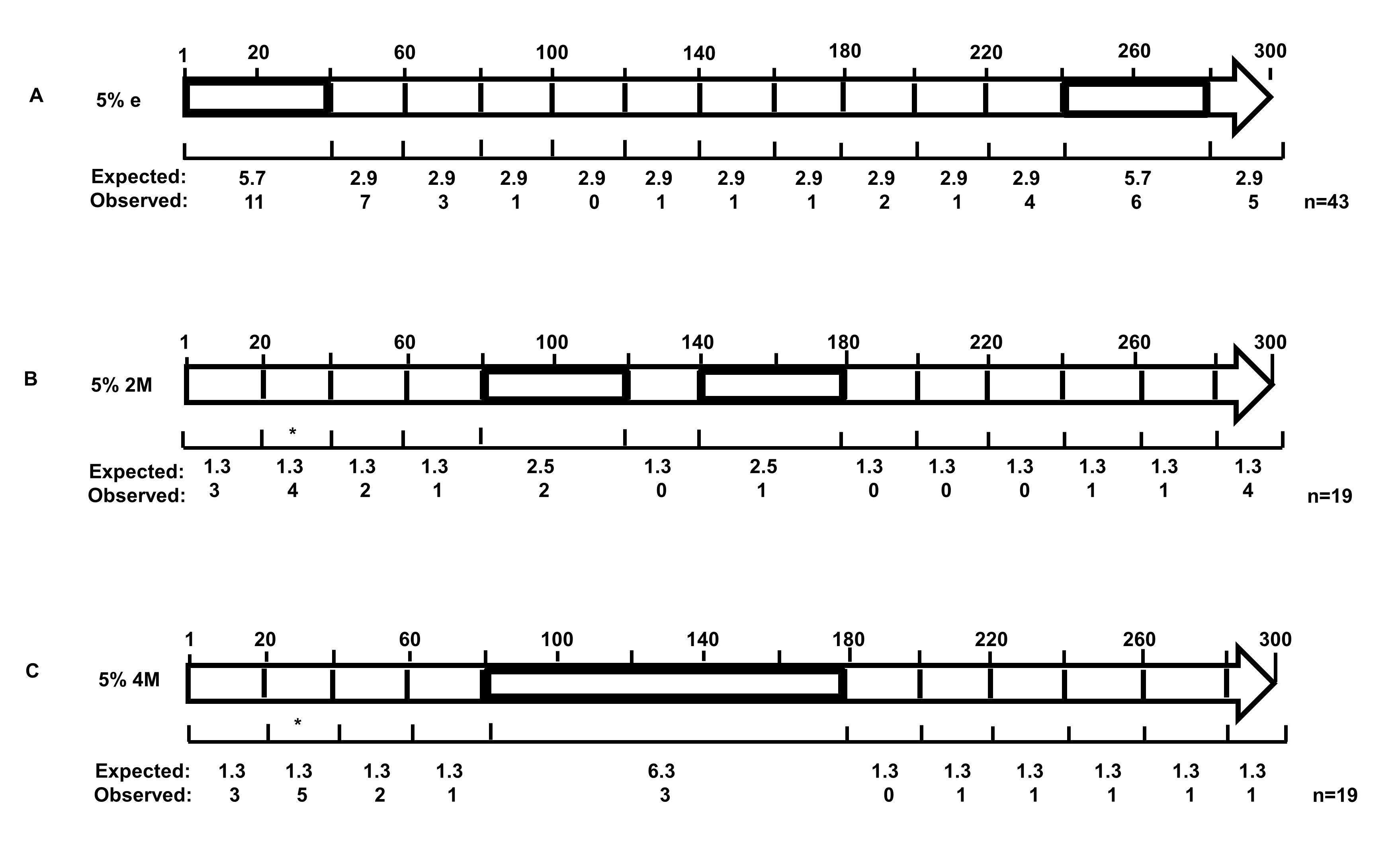

Supplement: S9 Fig — The Alu1 sequence in 5%-AARP was modified to include larger stretches of homology to Alu2 (Alu Ya5 consensus sequence) and subsequently inserted into HEK293FRT cells (see S1 Fig, Materials and Methods). (A) The distribution of Alu/Alu recombination junctions for 5%e-AARP (40 bp stretches of sequence homology at the 5’ and 3’ ends of Alu1) HEK293FRT cells was determined by PCR and sequence analysis of DNA repair products from isolated puror colonies. The Alu/Alu recombination product is divided into segments according to the intervals of homology in which the Alu/Alu recombination junction can be mapped. The number of Alu/Alu recombination junctions expected and observed in each interval is shown. While not statistically significant at p<0.05, there is still a slight preference for resolution of repair at the 5’ end of the Alu element. (B) The distribution of Alu/Alu recombination junctions for 5%2M-AARP (contains two stretches of 40 bp of sequence homology in the middle of Alu1) HEK293FRT cells was determined as described in (A). An asterisk (*) marks an interval in which p<0.05 significance as determined by a chi-square test for observed vs. expected. No increase in recombination within the stretches of homology was observed. In fact, we again observe a statistically significant bias for recombination resolution within the first 100 base pairs of the Alu element. (C) The distribution of Alu/Alu recombination junctions for 5%4M-AARP (contains 100 bp of sequence homology in the middle of Alu1) HEK293FRT cells was determined as described in (A). An asterisk (*) marks an interval in which p<0.05 significance as determined by a chi-square test for observed vs. expected. No significant increase in recombination within the stretch of homology was observed. Again, however, we observed a statistically significant increase in recombination repair junctions within the first 100 base pairs of the Alu element compared to the number expected based on a random distribution. (TIF) [file pgen.1005016.s009.tif]

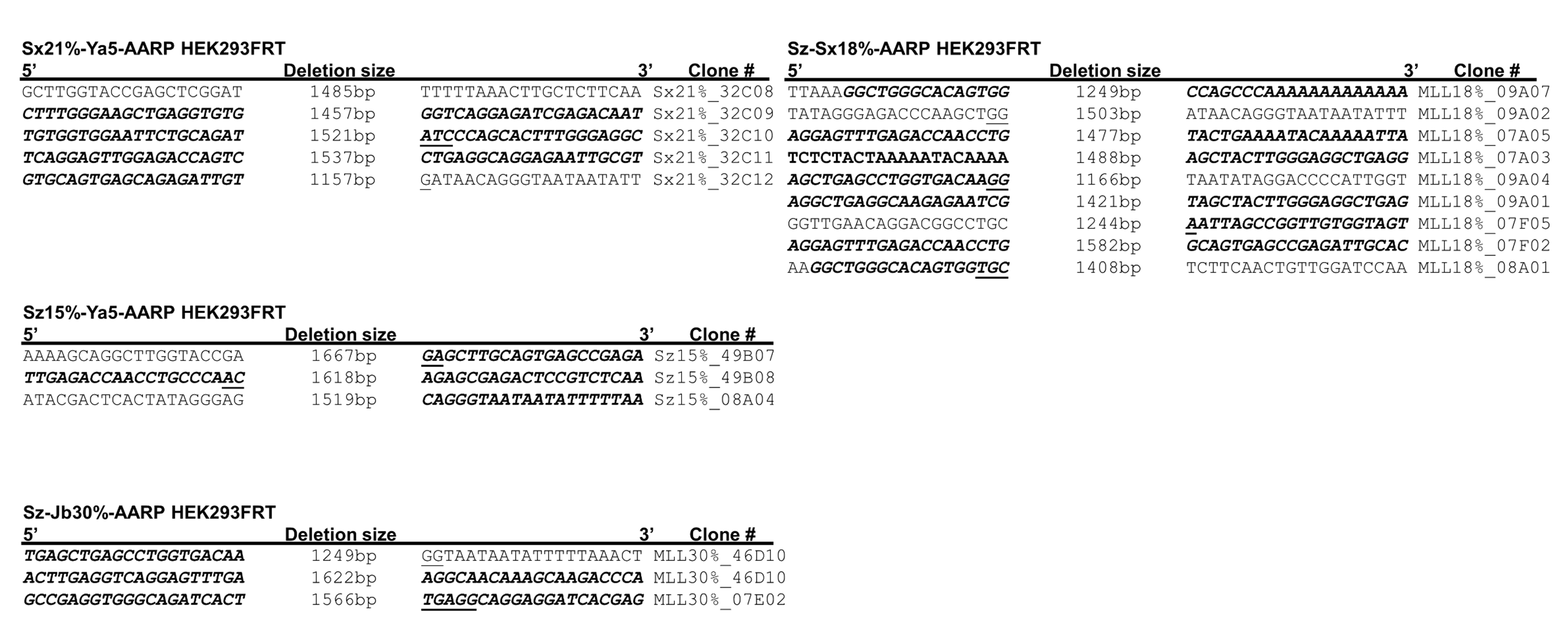

Supplement: S10 Fig — The NHEJ repair junctions recovered from isolated puror colonies are shown as the size of the variable deletions along with the sequences flanking the deletion on both sides. Deletions ranged in size from 897 base pairs to 1901 base pairs. Alu element sequences are in bold italics. Alu1 or flanking AARP reporter cassette sequence is shown to the left of the NHEJ deletion and Alu2 or flanking AARP reporter cassette sequence is shown to the right of the NHEJ deletion. Underlined sequences indicate a microhomology found at the NHEJ repair junction. (TIF) [file pgen.1005016.s010.tif]

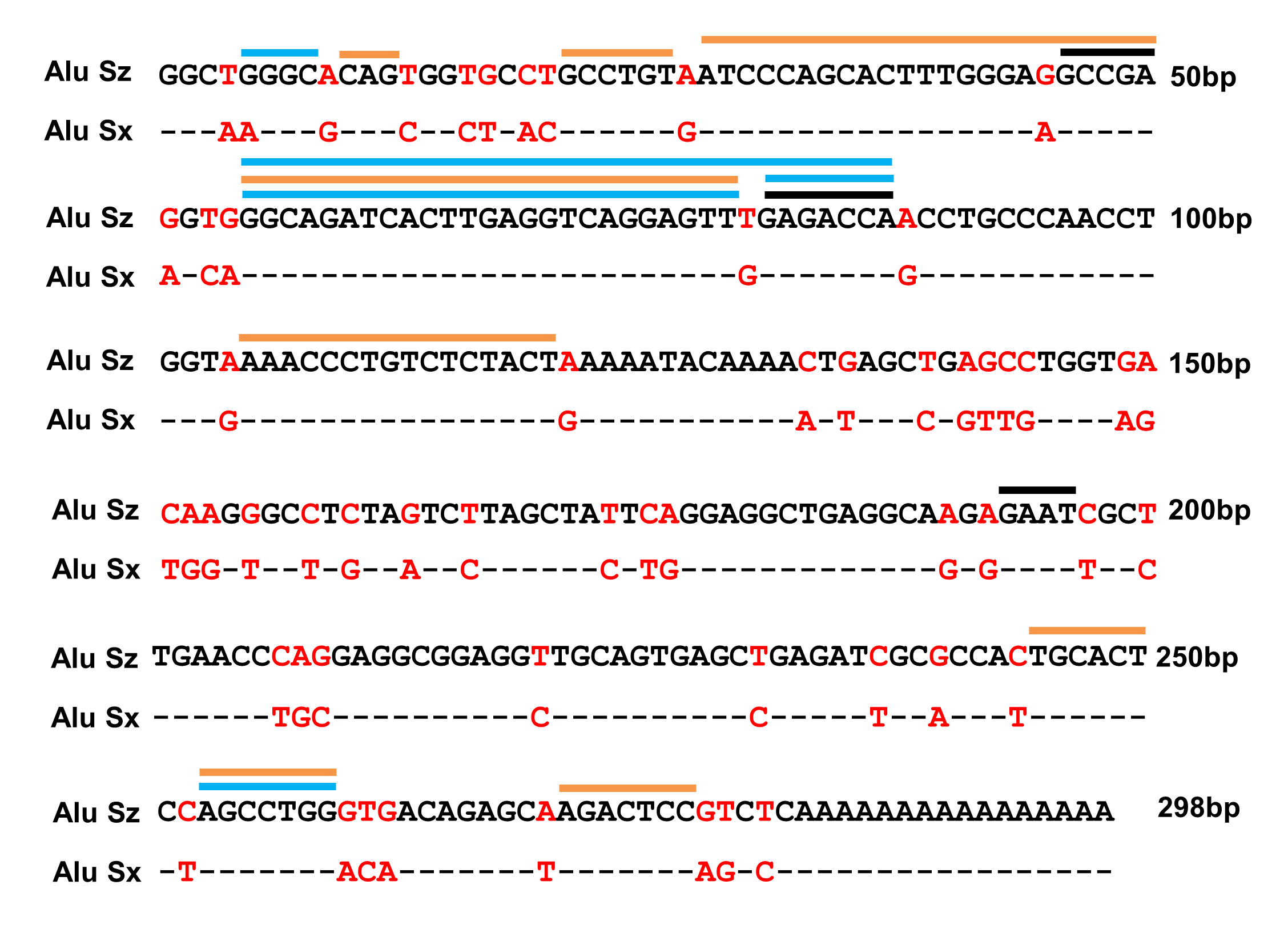

Supplement: S11 Fig — Black bars above the sequence represent the interval containing the Alu/Alu recombination junctions reported in this study. Similarly, blue bars represent those reported by [7] and orange bars those reported by [23,24]. (TIF) [file pgen.1005016.s011.tif]

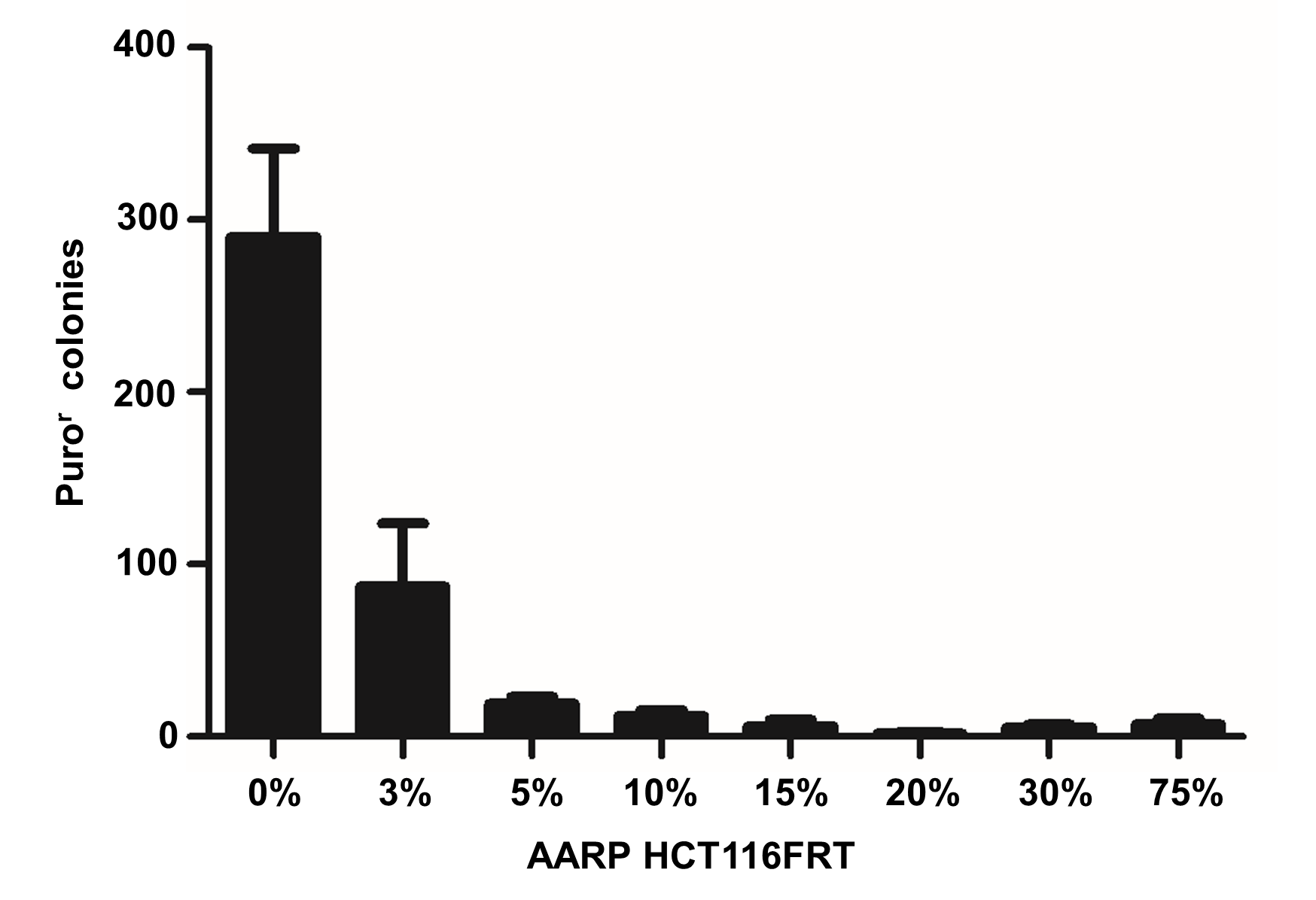

Supplement: S12 Fig — Stably integrated AARP HCT116 cell lines were generated using the Flp-In system (see S1 Fig, Materials and Methods) and transfected with I-SceI expression vector to induce DNA DSBs. The average number of puror colonies is plotted for the indicated AARP HCT116FRT cells tested. Data from at least three independent experiments using at least three independently isolated clones for each AARP cell line are averaged with error bars indicating standard error. (TIF) [file pgen.1005016.s012.tif]

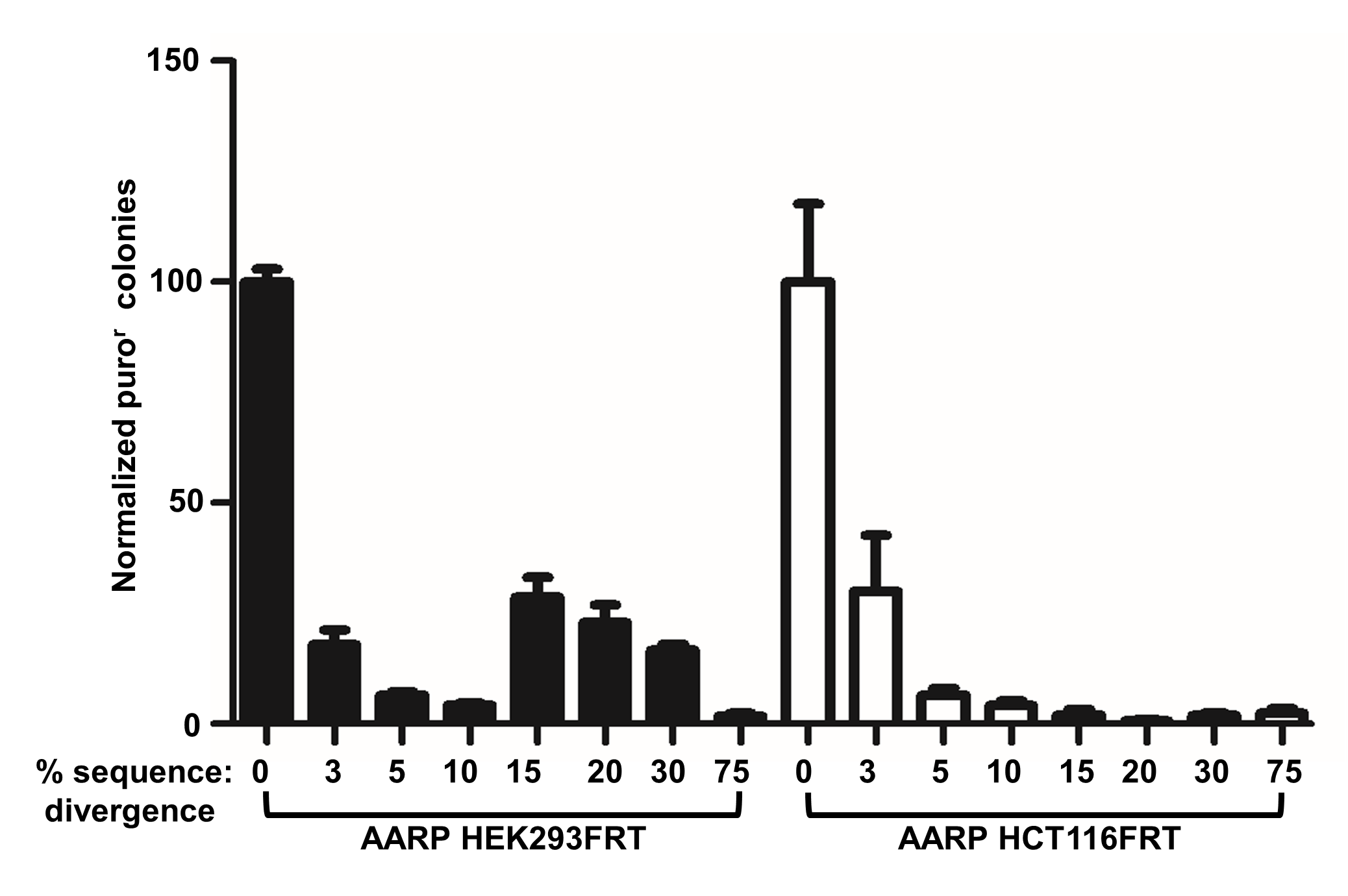

Supplement: S13 Fig — The average number of puror colonies for AARP HEK293FRT cells (Fig. 2B and Table 1) and AARP HCT116FRT cells (S12 Fig) is shown with 0%-AARP colony numbers normalized to 100. Data from at least three independent experiments using at least three independently isolated clones for each AARP cell line are averaged with error bars indicating standard error. AARP HCT116FRT cells lack the enhancement of puror colony formation within the 15–30% Alu element sequence divergence as observed with HEK293FRT cells. (TIF) [file pgen.1005016.s013.tif]

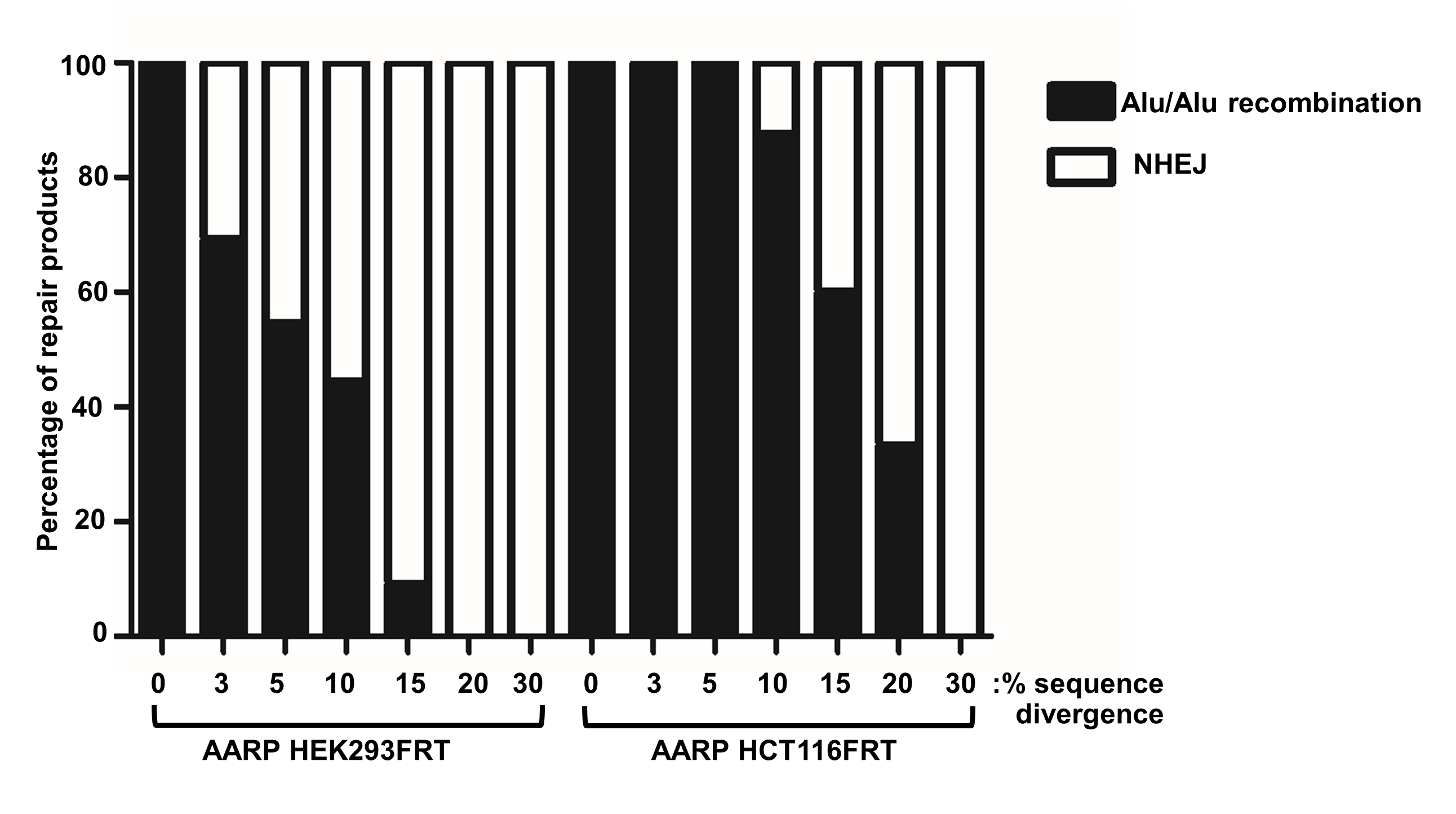

Supplement: S14 Fig — The percentage of Alu/Alu recombination or NHEJ as determined by PCR and sequence analysis of DNA repair products from isolated puror colonies is shown. (TIF) [file pgen.1005016.s014.tif]

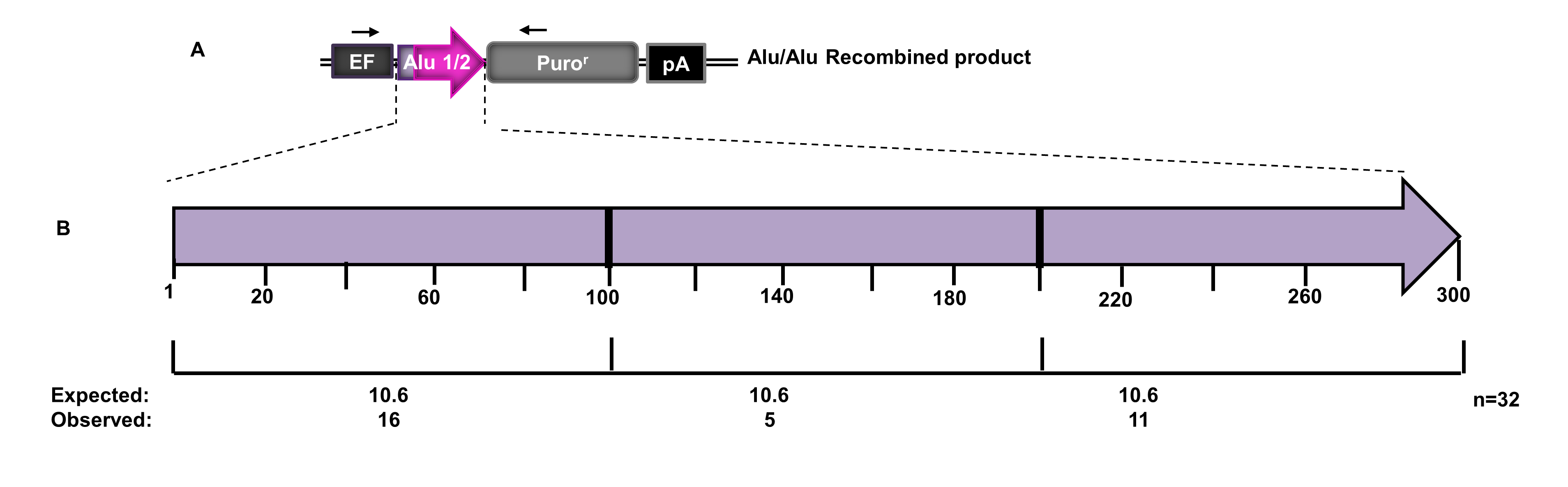

Supplement: S15 Fig — (A) A schematic of the Alu/Alu recombination product as shown in Fig. 3A. (B) The distribution of Alu/Alu recombination junctions in diverged AARP HCT116FRT cells as determined by PCR and sequence analysis of DNA repair products from isolated puror colonies. The Alu/Alu recombination product is divided into three segments of equal length (100 bp), which each contain the same extent of sequence divergence. The number of Alu/Alu recombination junctions expected and observed in each 100 bp interval is shown. (TIF) [file pgen.1005016.s015.tif]

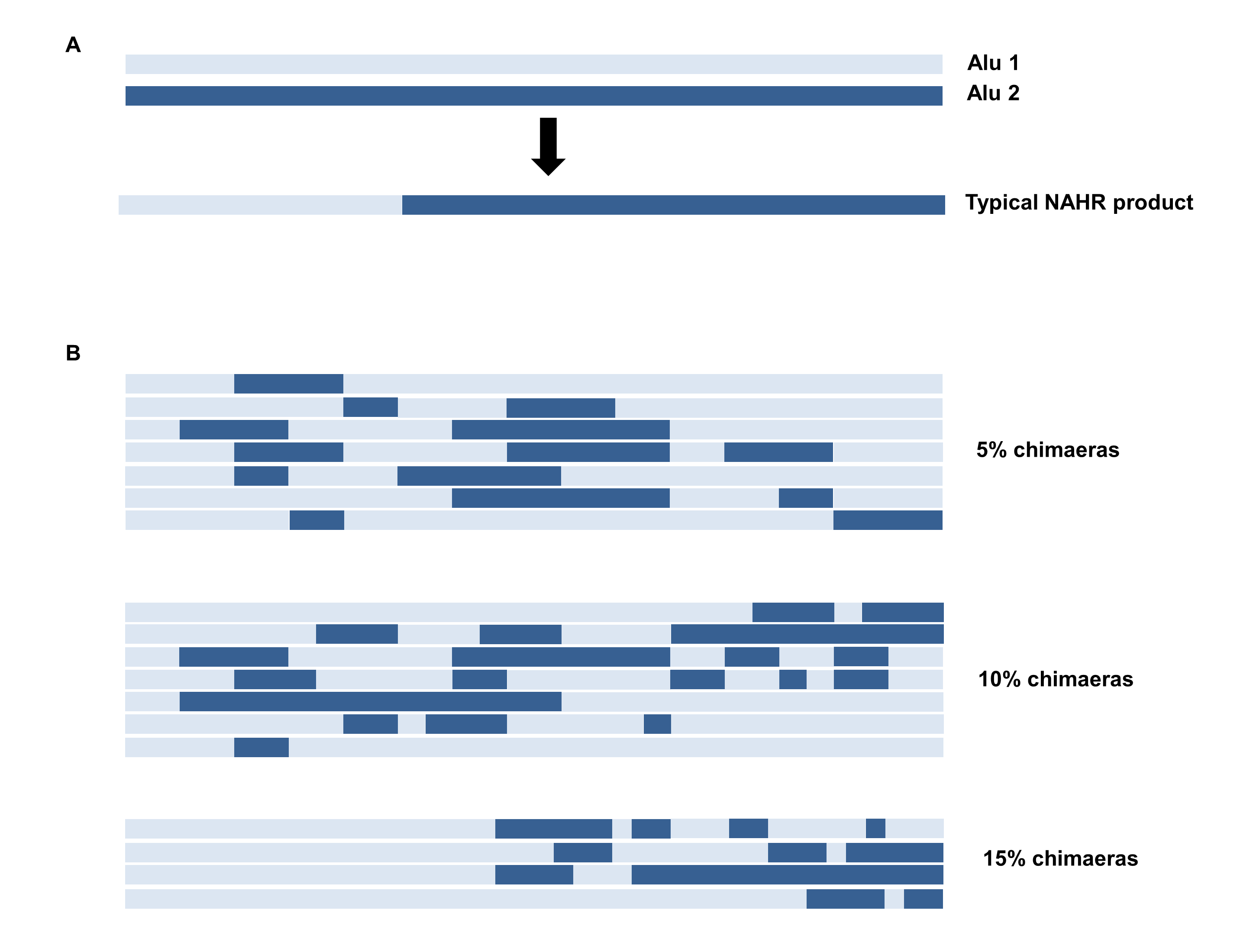

Supplement: S16 Fig — (A) Schematic showing the typical Alu/Alu recombination product observed. The majority of intact Alu elements show a single conversion location within the Alu element, which implicates MMEJ as the repair pathway used for the majority of Alu/Alu recombination events between diverged Alu elements. (B) Schematics showing the complex Alu element complex chimeras generated in a minority of Alu/Alu recombination events in 5%-, 10%-, and 15%-AARP HEK293FRT cells. Multiple conversion locations can be observed in these Alu/Alu recombination events, which implicates repair of mismatched tracts within a SSA heteroduplex intermediate to generate “patches” of Alu1 and Alu2 in each AARP clone characterized. (TIF) [file pgen.1005016.s016.tif]

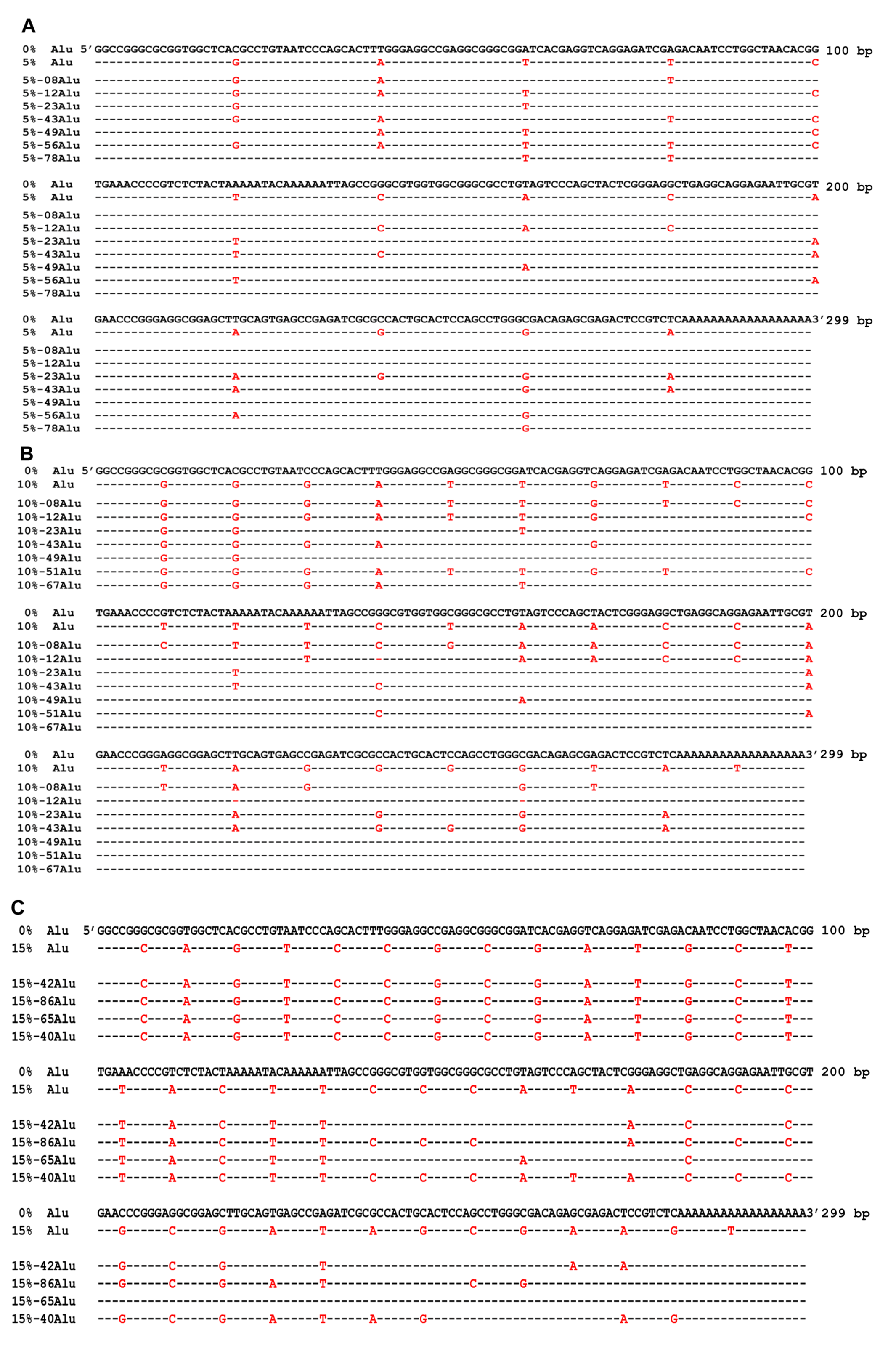

Supplement: S17 Fig — Sequence alignments of the complex chimeras generated during Alu/Alu recombination events described schematically in S17 Fig are shown for (A) 5%-AARP, (B) 10%-AARP, and (C) 15%-AARP HEK293FRT cells. (TIF) [file pgen.1005016.s017.tif]

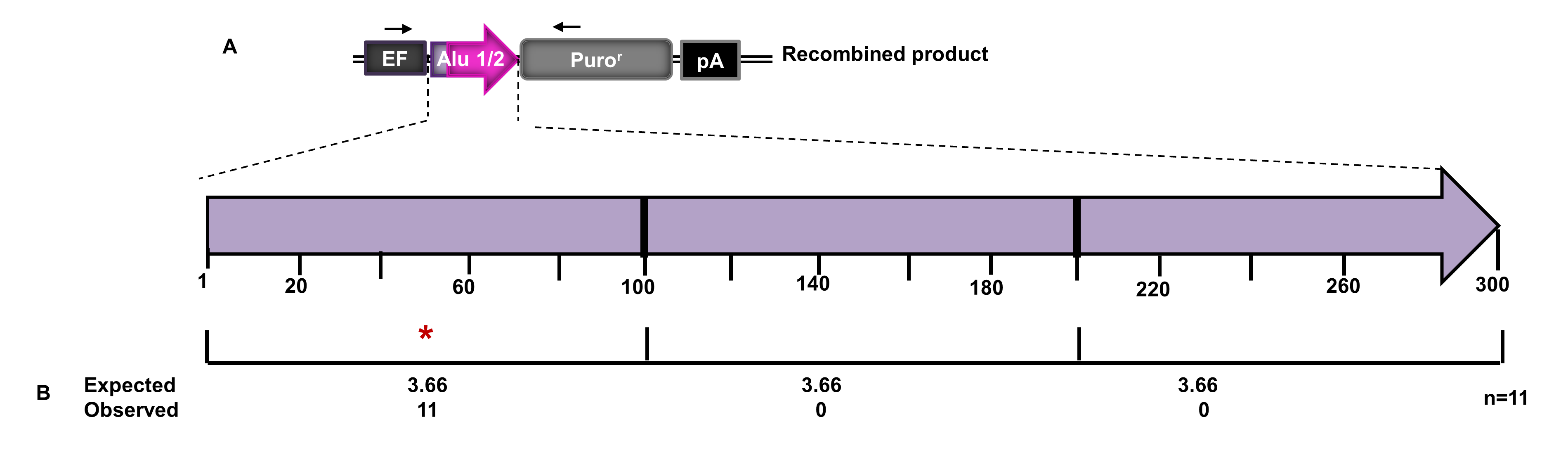

Supplement: S18 Fig — (A) A schematic of the Alu/Alu recombination product as shown in Fig. 3A. (B) The distribution of Alu/Alu recombination junctions in indicated AARP HEK293FRT cells treated with Rad52 siRNAs as determined by PCR and sequence analysis of DNA repair products from isolated puror colonies. The Alu/Alu recombination product is divided into three segments of equal length (100 bp), which each contain the same extent of sequence divergence. The number of Alu/Alu recombination junctions expected and observed in each 100 bp interval is shown. An asterisk (*) marks an interval in which p<0.05 significance as determined by a chi-square test for observed vs. expected. (TIF) [file pgen.1005016.s018.tif]

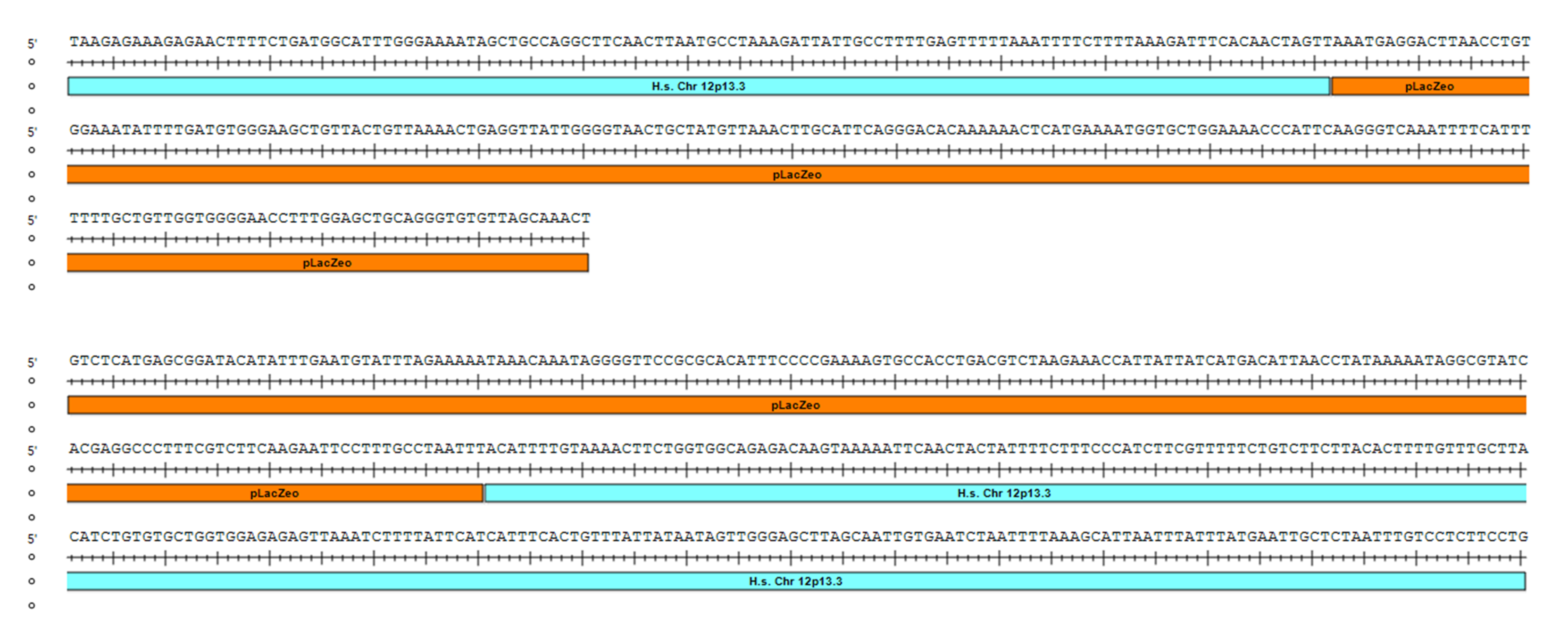

Supplement: S19 Fig — Both sequence junctions of the integration of the pFRT/lacZeo vector at Chr 12 in HEK293FRT cells (HEK293-based Flp-In cells; Life Technologies) are shown as determined by whole genome sequencing with Illumina Next Generation Sequencing. (TIF) [file pgen.1005016.s019.tif]
