# Supplementary material for: The Contribution of Alu Elements to Mutagenic DNA Double-Strand Break Repair
Source: PLoS Genet. 2015 Mar 11;11(3):e1005016. doi: 10.1371/journal.pgen.1005016 (PMC4356517; doi:10.1371/journal.pgen.1005016)
Supplement: S1 Table — The names incorporate the percent divergence between Alu2 (Ya5 consensus) and the variant Alu1 placed in most of the vectors. Most of the actual Alu1 sequences for these vectors are shown in S4 Fig. The 5%e represents evenly spaced mutations every 20 bp but with the ends having twice the length of consensus sequence match. The 5%2M and 5%4M in names refers to a stretch of either 80 or 100 bp of consensus sequence match, respectively, located in the center of the Alu. The locations of these longer homology stretches are shown schematically in S9 Fig. The Scr1- and Scr2-AARP names refer to two versions of totally randomized sequence replacing Alu1 (75%). The Sz, Sx and Jb nomenclature refers to natural Alu elements that are described in the Results. (DOCX) [file pgen.1005016.s020.docx]

**Supplementary Table 1: Homeologous recombination vectors used in this study**

| **Name of the homeologous recombination vector** | **Alu replaced in the recombination vector** | **Distribution of divergence** |
| --- | --- | --- |
| 0.7%-AARP | Alu1 | N/A |
| 3%-AARP | Alu1 | Evenly spread (every 40bp) |
| 5%-AARP | Alu1 | Evenly spread (every 20bp) |
| 5%e-AARP | Alu1 | Evenly spread (every20bp)  40bp of homology at the ends |
| 5%2M-AARP | Alu1 | Evenly spread (every20bp)  80bp of homology in the middle |
| 5%4M-AARP | Alu1 | Evenly spread (every20bp)  100bp of homology in the middle |
| 10%-AARP | Alu1 | Evenly spread (every 10bp) |
| 15%-AARP | Alu1 | Evenly spread (every 6-7bp) |
| 20%-AARP | Alu1 | Evenly spread (every 5bp) |
| 30%-AARP | Alu1 | Evenly spread (every 2-3bp) |
| 75%(1)-AARP | Alu1 | N/A |
| 75%(2)-AARP | Alu1 | N/A |
| Sz15%-AARP | Alu1 | Random |
| Sx21%-AARP | Alu1 | Random |
| MLL Sz-Sx18%-AARP | Alu1-Alu2 | Random |
| MLL Sz-Jb30%-AARP | Alu1-Alu2 | Random |

**N/A: not applicable**
