# Supplementary material for: The Contribution of Alu Elements to Mutagenic DNA Double-Strand Break Repair
Source: PLoS Genet. 2015 Mar 11;11(3):e1005016. doi: 10.1371/journal.pgen.1005016 (PMC4356517; doi:10.1371/journal.pgen.1005016)
Supplement: S2 Table — (DOCX) [file pgen.1005016.s021.docx]

**Supplementary Table 2: Oligonucleotides Used in this Study**

| **AARP Rescue primers** | **Sequence** |
| --- | --- |
| EF1 FP | 5’ GAGAATCGGACGGGGGTAGT 3’ |
| Puro RP | 5’ CGCTGGTCTCCAGGAAGG 3’ |
| AARP Sequencing primer | 5’ GGTCACGCGTTCAATGTGG 3’ |
