# Supplementary material for: The Contribution of Alu Elements to Mutagenic DNA Double-Strand Break Repair
Source: PLoS Genet. 2015 Mar 11;11(3):e1005016. doi: 10.1371/journal.pgen.1005016 (PMC4356517; doi:10.1371/journal.pgen.1005016)
Supplement: S3 Table — The results of this experiment are shown in S2 Fig. (DOCX) [file pgen.1005016.s022.docx]

**Supplementary Table 3: ddPCR Primers and probes**

| **Primer/Probe Name** | **Sequence** |
| --- | --- |
| Neo Probe | 5’ 6FAM CCGCCGTGTTCCG BHQ1 3’ |
| Neo Fwd | 5’ CAGCAGACCATCGGGTGTAG 3’ |
| Neo Rev | 5’ CAGGTCGGTCTTCACGAACA 3’ |
