# Supplementary material for: The Contribution of Alu Elements to Mutagenic DNA Double-Strand Break Repair
Source: PLoS Genet. 2015 Mar 11;11(3):e1005016. doi: 10.1371/journal.pgen.1005016 (PMC4356517; doi:10.1371/journal.pgen.1005016)
Supplement: S4 Table — In each experiment 106 cells are transfected with control (No DSBs) or I-SceI expression plasmid. Because no replating occurs before colony formation, the number of repair events measured by puror colonies directly correlates with the repair frequency and it has been adjusted to frequency per 100,000 cells for more direct comparison with other studies and given with standard error. (DOCX) [file pgen.1005016.s023.docx]

**Supplementary Table 4: DSB-induced repair frequency**

| **Alu sequence divergence in AARP** | **No DSB puro^r^ frequency (x10^-5^)^a^** | **DSB-Induced (I-SceI) puro^r^ frequency (x10^-5^)^a^** |
| --- | --- | --- |
| 0% | 0.8 (±0.1) | 116.4 (±3.4) |
| 0.7% | 0 | 82.2 (±2.5) |
| 3% | 0 | 20.9 (±3.9) |
| 5% | 0 | 7.7 (±0.8) |
| 10% | 0 | 5.0 (±0.6) |
| 15% | 0 | 33.4 (±5.2) |
| 20% | 0 | 26.7 (±4.6) |
| 30% | 0 | 19.5 (±1.6) |
| 75% | 0 | 2.1 (±0.6) |

^a^ The frequency of puro^r^ colonies was determined using the number of 1 x 10^6^ for cells plated for transfection.
